# Transcriptional profiling to identify the key genes and pathways of pterygium

**DOI:** 10.7717/peerj.9056

**Published:** 2020-05-04

**Authors:** Yihui Chen, Haoyu Wang, Yaping Jiang, Xiaoyan Zhang, Qingzhong Wang

**Affiliations:** 1 Department of Ophthalmology, Yangpu Hospital, Tongji University School of Medicine, Shanghai, China; 2 Department of Dermatology, Shanghai Ninth People’s Hospital, Shanghai Jiao Tong University School of Medicine, Shanghai, China; 3 Department of Ophthalmology, Huashan Hospital, Fudan University, Shanghai, China; 4 Shanghai Key Laboratory of Compound Chinese Medicines, The MOE Key Laboratory for Standardization of Chinese Medicines, Institute of Chinese Materia Medica, Shanghai University of Traditional Chinese Medicine, Shanghai, China

**Keywords:** RNA sequencing, Pterygium, Weighted gene co-expression network analysis, Hub genes, Ocular surface disease, Pathogenesis

## Abstract

**Purpose:**

Pterygium results from a variety of biological pathways that are involved in the formation of ocular surface diseases. However, the exact pathogenesis of pterygium is still unclear. Our study focused on gene expression profiles to better understand the potential mechanisms of pterygium.

**Methods:**

RNA sequencing experiments were performed on clinical pterygium tissues and normal conjunctival tissues. To identify the hub genes for the development of pterygium, we further conducted weighted gene co-expression network analysis (WGCNA). qRT-PCR was utilized to validate the dysregulation of the most significant differentially expressed genes (DEGs) and key hub genes in the independent subjects.

**Results:**

A total of 339 DEGs (P-adjusted < 0.05 and log2 fold change [log2FC] ≥ 1.0) were obtained that reached statistical significance with p-values < 0.05. Among them, 200 DEGs were upregulated; these genes were mainly associated with the extracellular matrix and with cell adhesion or migration. In contrast, the 139 downregulated genes were enriched for endocrine and inflammation pathways. With regard to WGCNA, five modules were assigned based on the DEG profiles, and the biological functions of each module were verified with previously published GO terms. The functions included ECM-receptor interactions, the PI3K-Akt signalling pathway and an endoplasmic reticulum (ER)-related pathway. The five hub genes with the highest connectivity in each module and the five most significant DEGs showed dysregulated expression in the independent cohort samples.

**Conclusions:**

RNA sequencing and WGCNA provided novel insights into the potential regulatory mechanisms of pterygium. The identified DEGs and hub genes, which were classified into two groups according to different functions or signalings, may provide important references for further research on the molecular biology of pterygium.

## Introduction

Pterygium, a common ocular surface disease, is mainly characterized by excessive proliferation of fibrovascular tissue from the conjunctiva to the cornea (Chui et al., 2011). At present, the standard treatment for pterygium is surgical removal, but the recurrence rate is approximately 61–82% after this treatment (Sheppard et al., 2014; Singh, 2017). Epidemiological studies have suggested that environmental factors, including ultraviolet radiation and dust, are involved in the etiology of pterygium (Zhou et al., 2016). Recent molecular studies have also reported that biological pathways including angiogenesis, fibrosis, proliferation and inflammation play contributory roles in the development of pterygium (Larrayoz et al., 2012). In general, the pathology of pterygium involves multiple factors. However, the exact pathogenesis remains unclear (Serra et al., 2018).

Recently, increasing studies on pterygium have focused on transcriptional analyses and have reported the existence of some differentially expressed genes (DEGs) in pterygium patients. Using a DNA microarray experiment, Wong et al. (2006) found that the mRNA levels of a number of genes were altered in primary pterygium. It was noted that the IGFBP3 gene, the function of which is related to the effects of insulin-like growth factor on cells, was significantly decreased in pterygium. Subsequently, other groups have also performed transcriptional profiling studies on pterygium (Lan et al., 2018; Liu et al., 2016; Liu et al., 2017).

RNA sequencing (RNA-Seq) technology, a novel transcriptional profiling tool, has several advantages over other techniques, particularly its sensitivity in terms of measuring gene expression and its ability to detect dynamic changes. With the rapid development of high throughput next generation sequencing (NGS), the discovery of disease-associated genetic variants and genome-wide profiling of expressed sequences and epigenetic marks has become more intensive, thereby permitting systems-based analysis of ocular development and diseases, including pterygium (Chaitankar et al., 2016; Larrayoz et al., 2012). Kim et al. (2016) assessed that the role of neural retina leucine zipper (NRL) in transcript development of rod photoreceptors and its relationship with other transcriptional regulators and effectors by performing microarray hybridization and RNA-Seq on mouse retinal tissues (Kim et al., 2016). Subsequently, another RNA sequence was performed on developing mouse rod photoreceptors in retinal tissues, indicating that NRL could regulate the noncoding transcriptome in developing photoreceptors (Zelinger et al., 2017). Recently, Bang et al. utilized RNA-Seq to identify the expression of complement factors in 20 cases of pterygium and in normal conjunctival tissues. The researchers reported that pterygium size is related to the expression of CFH, C1QB, C1QC and MASP1 genes and the alternative and lectin-binding complement systems may be activated in diseased tissues (Bang et al., 2017). In that study, novel DEGs and potential mechanisms of pterygium were mined from whole transcriptome profiles. Overall, studies on pterygium using RNA-Seq are still relatively scarce; thus, more studies with larger sample sizes are needed.

In the current study, we did the following: (a) explored the transcriptional profiles of pterygium with RNA-Seq; (b) constructed a weighted co-expression network; (c) identified the key hub mRNAs significantly associated with pterygium; and (d) raised new potential mechanisms associated with pterygium.

## Materials and Methods

### Patients and specimens

The research protocol was approved by the Institutional Review Board of Yangpu Hospital, and all study participants gave written informed consent. The study was carried out in accordance with the Declaration of Helsinki. All surgical procedures were performed under local anesthesia by the same surgeon. Thirty patients underwent elective pterygium surgery, including 18 males (aged 56 to 77 years, mean age 67 years) and 12 females (aged 40 to 77 years, mean age 62 years). Control tissues, i.e., small rectangular pieces of normal conjunctival tissue, were excised from 32 donor eyes, which were matched for age (42 to 74 years, mean age 64 years), gender (18 males and 14 females), and ethnic background (Chinese). Eight pterygium tissues and 10 normal conjunctival tissues were subjected to RNA-Seq. Ten pterygium tissues and 10 normal conjunctival tissues were used for validation of hub gene expression. To verify the dysregulation of the top five transcripts from the RNA-Seq data, we selected 12 independent pterygium and 12 healthy control samples to examine the mRNA levels. All study participants with pterygia were subjected to slit lamp photography (Canon, Japan) preoperatively to demonstrate the ingrowth of the pterygium onto the cornea. The extension of the pterygium onto the cornea in patients ranged from two mm to four mm. Clinical surgery for pterygium involved conventional excision of the pterygium with autotransplantation of the conjunctiva. The collected samples were transferred to 1.5 ml tubes and stored at −80 °C for further analysis.

### RNA-Seq

Total RNA was extracted from the frozen tissues with TRIzol reagent (Invitrogen, CA, US) according to the manufacturer’s protocol. The RNA concentration and purity were measured with a NanoDrop ND2000 (Thermo Scientific, MA, USA). The integrity of the total RNA was examined with an Agilent Bioanalyzer 2100 (Agilent Technologies, CA, US). The 260 nm/280 nm ratio was required to be within the range 1.8–2.2, and the RNA integrity number was required to be higher than 7 for the RNA-Seq experiments.

Two micrograms of mRNA were prepared for construction of the RNA-Seq library. First, we removed the ribosomal RNA from the total RNA with an Epicentre Ribo-Zero™ rRNA Removal Kit (Epicentre, WI, USA). Briefly, we hybridized probes to the RNA, and then the mixture was digested with RNase H and DNase I. The RNA was purified with an Agencourt RNAClean XP system. The sequencing libraries were constructed using a NEBNext^®^ Ultra™ Directional RNA Library Prep Kit for Illumina^®^ (NEB, MA, USA). The purified mRNA was fragmented and reverse-transcribed into first-strand cDNA with random hexamers in the presence of actinomycin D. Then, the second-strand cDNA was synthesized with RNase H and DNA polymerase I. After purification, the cDNA was subsequently subjected to adaptor ligation, USER enzyme digestion and PCR library enrichment. The final purified library was measured and quantified with the Bioanalyzer 2100 (Agilent, CA, USA) and sequenced on an Illumina HiSeq 2500 platform.

### RNA-Seq data processing and analysis

We utilized the software FastQC to assess the quality of the Illumina reads, which were trimmed with the FASTX-Toolkit. Then, the human assembly GRCh37 was downloaded from the Ensembl database; indexing was conducted with bowtie2, and the quality trimmed reads were mapped to the genome using TopHat (v 2.0.9). HTSeq was used to compute the read counts for each gene in each sample. The data of DEGs was normalized using the transfer matrix method (TMM). Next, the DEGs were screened with the software DESeq2. The *p*-values were adjusted with the Benjamini–Hochberg method for multiple comparison testing. The significance of DEGs was accepted at an adjusted *p*-value lower than 0.05.

### Gene Ontology and enrichment analysis

To illustrate the biological functions and pathways of the DEGs, we conducted Gene Ontology (GO) and pathway enrichment analyses. The topGO and clusterProfiler packages in R were utilized to detect GO category enrichment and Kyoto Encyclopedia of Genes and Genomes (KEGG) pathway overrepresentation from the entire database. Adjusted *p*-values were calculated with the Benjamini–Hochberg method, and the terms with *p*-values <0.05 were considered to be significant.

### Weighted gene co-expression network analysis

To further delineate the functions of the DEGs, we conducted weighted gene co-expression network analysis on the basis of DEG expression in the studied tissues. According to the WGCNA tutorial, the step-by-step network construction approach was used for module identification. Firstly, we selected the suitable soft-thresholding power by testing a set of candidate powers to evaluate the approximate scale-free topology. Subsequently, the soft-thresholding power equal to 10 was used for calculation of the adjacencies. Then, the adjacencies were transformed into a topological overlap matrix for obtaining the corresponding dissimilarity. The *hclust* function was applied to produce a hierarchical clustering tree of genes. The leaves in the clustering trees corresponds to individual genes, while branches of the clustering trees represent the highly interconnected and co-expressed genes. The package *dynamicTreeCut* function for branch cutting, which has the advantages of identifying modules that have highly similar gene expression signatures, was used to detect modules in which the genes were highly co-expressed in the dendrogram groups. After that, module trait association analysis was used to find correlations between modules and phenotypes. Then, the function *TopHubInEachModule* was used to identify the hub gene in each module that had high connectivity in the weighted co-expression network. The figures were created using the *igraph* package. Finally, to characterize the heterogeneity of gene expression patterns quantitatively, the personalized expression perturbation profiles (PEEPs) algorithm was performed to identify expression changes within each individual.

### Validation of the mRNA expression of hub genes by quantitative real-time PCR

Based on the RNA-seq results, we chose LCN1 (lipocalin 1), LTF (lactotransferrin), SCGB2A1 (secretoglobin family 2A member 1), HBA1 (hemoglobin subunit α1) and HBA2 (hemoglobin subunit α2), which were the top five DEGs in the comparison between pterygium and normal tissues. As well as the five hub genes identified from five modules in line with WGCNA analysis, including FN1 (fibronectin 1), ECM1 (extracellular matrix protein 1), GADD34 (growth arrest and DNA damage inducible gene 34), CXCL12 (C-X-C motif chemokine ligand 12) and IQGAP2 (IQ motif containing GTPase activating protein 2) for qRT-PCR validation.Total mRNA from each sample was isolated using TRIzol (Life Technologies, CA, USA), and the purity ratios (260/280 nm and 260/230 nm) were assessed with a NanoDrop instrument (Thermo Scientific, MA, USA). SYBR Premix Ex Taq II (Takara, Shiga, Japan) and synthesized primers sets (Sangon, Shanghai, China) were used for qRT-PCR. The methods for qRT-PCR were followed as described in our earlier publication (Wang et al., 2018). GAPDH was used as the endogenous control gene. The fold changes were calculated using the 2^−ΔΔ*Ct*^method.

### Statistical analysis

Data analysis was performed with the R platform and GraphPad Prism. The data are presented as the mean ± SEM. The pterygium and control groups were compared using independent-sample *t*-tests, and the significance was set at *p* ≤ 0.05.

## Results

### Identification of the DEGs

**Figure 1:**
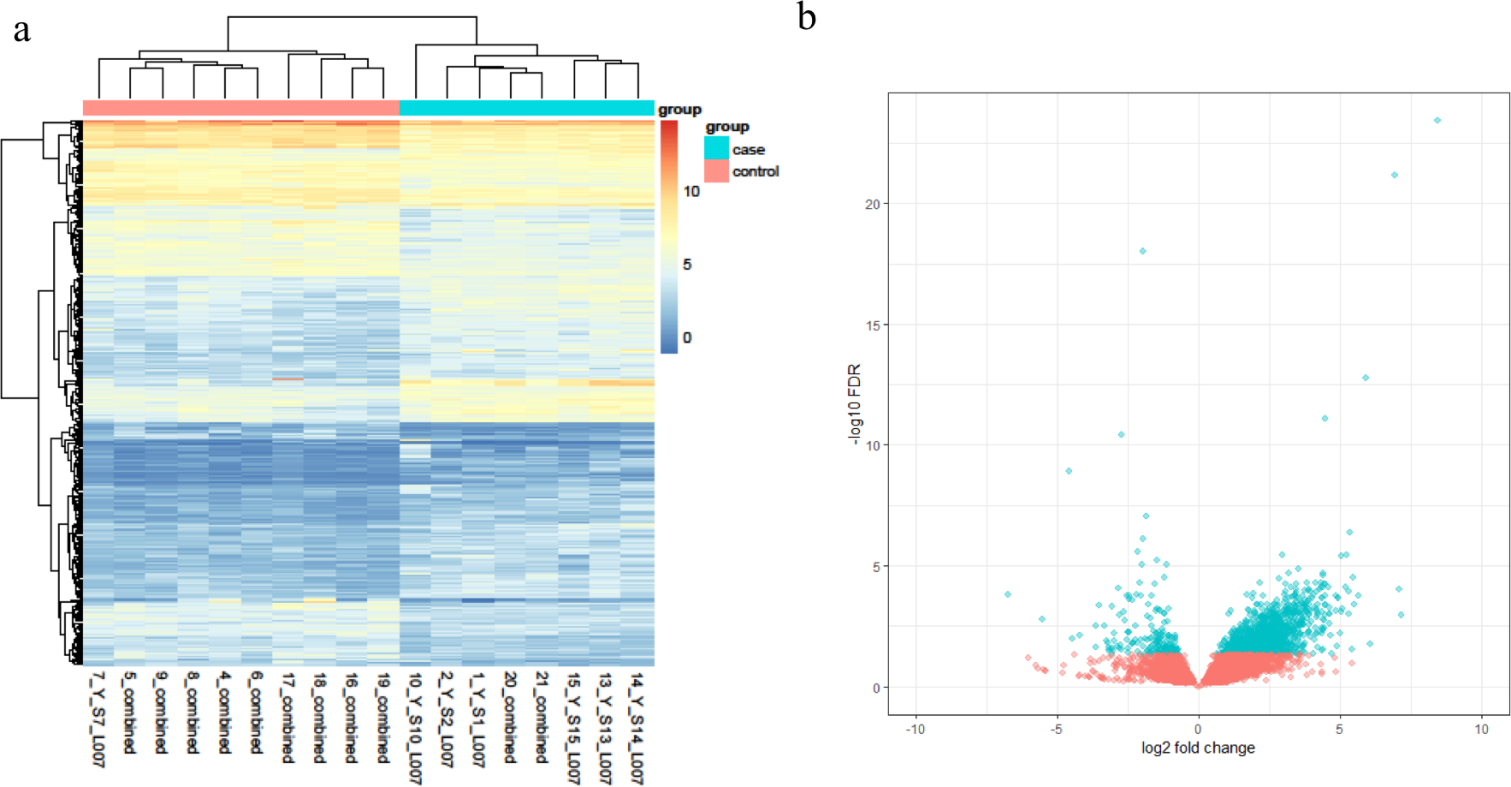
(A) The heatmap plot of differentially expressed genes in each subject; (B) the volcano plot of the 339 DEGs genes.

Based on the RNA-Seq data, a total of 339 DEGs were obtained, and these genes reached the threshold *p*-value of <0.05. Among these DEGs, 200 transcripts were upregulated in the disease group against the health one, while 139 transcripts were found to decrease in pterygium tissues. The top 10 genes with the most obvious expression changes based on adjusted *p*-values are listed in Table 1. In addition, the relative expression levels are shown in a heatmap plot (Fig. 1A) and a volcano plot (Fig. 1B).

**Table 1:** The top 10 adjusted p-value up-regulated and down-regulated genes.

**Gene**	**Description**	**Gene ID**	**Regulation**	**Base mean**	**Fold change**	**Lfc SE**	**Stat**	***P*-value**	**Padj**
HBA1	hemoglobin subunit alpha 1	3039	Up	224.696	6.4604	0.6070	10.6437	1.87E−26	1.44E−22
HBB	hemoglobin subunit beta	3043	Up	310.551	6.2194	0.5831	10.6657	1.47E−26	1.44E−22
SFRP4	secreted frizzled related protein 4	6424	Up	21.962	4.3063	0.5746	7.4946	6.65E−14	1.71E−10
FN1	fibronectin 1	2335	Up	101.513	3.2479	0.4457	7.2865	3.18E−13	7E-10
HBA2	hemoglobin subunit alpha 2	3040	Up	351.040	6.0738	0.8777	6.9201	4.51E−12	8.69E−09
HMCN1	hemicentin 1	83872	Up	20.280	2.4963	0.4078	6.1211	9.29E−10	0.000000937
ALAS2	5′-aminolevulinate synthase 2	212	Up	4.992	5.3951	0.9013	5.9863	2.15E−09	0.00000194
ADAMTSL3	ADAMTS like 3	57188	Up	12.724	2.3096	0.4167	5.5433	2.97E−08	0.0000241
MYADM	myeloid associated differentiation marker	91663	Up	108.143	2.3998	0.4425	5.4239	5.83E−08	0.0000374
PTPRB	protein tyrosine phosphatase, receptor type B	5787	Up	16.282	1.9619	0.3643	5.3856	7.22E−08	0.0000428
NR1D1	nuclear receptor subfamily 1 group D member 1	9572	Down	139.202	−4.0960	0.4400	−9.3092	1.29E−20	6.61E−17
BHLHE40	basic helix-loop-helix family member e40	8553	Down	382.273	−1.9441	0.2195	−8.8563	8.27E−19	3.18E−15
DUSP1	dual specificity phosphatase 1	1843	Down	612.930	−2.8342	0.3633	−7.8015	6.12E−15	1.88E−11
JUNB	JunB proto-oncogene, AP-1 transcription factor subunit	3726	Down	474.866	−1.9225	0.3003	−6.4015	1.54E−10	0.000000263
RARRES1	retinoic acid receptor responder 1	5918	Down	59.449	−2.3089	0.3630	−6.3612	2E-10	0.00000028
HIST1H1E	histone cluster 1 H1 family member e	3008	Down	219.832	−2.1612	0.3390	−6.3746	1.83E−10	0.00000028
ELF3	E74 like ETS transcription factor 3	1999	Down	346.545	−2.0415	0.3229	−6.3221	2.58E−10	0.000000331
ZFP36	ZFP36 ring finger protein	7538	Down	608.443	−2.0608	0.3296	−6.2527	4.03E−10	0.000000478
BCL6	BCL6, transcription repressor	604	Down	148.632	−1.7033	0.2745	−6.2049	5.47E−10	0.000000602
ADM	adrenomedullin	133	Down	31.998	−3.1517	0.5155	−6.1137	9.74E−10	0.000000937

### Gene function analysis for the upregulated genes

Using the software topGO and clusterProfiler, we performed GO and KEGG pathway enrichment analysis to find potential biological pathways of interest. Functional annotation of the 200 upregulated genes revealed that genes with higher expression levels were mainly involved in two biological functions: one was related to the extracellular matrix (ECM) and the other was related to cell adhesion or migration (Table 2). The top 50 GO terms, including biological process, cellular component and molecular process terms, are shown in Table S1.

**Table 2:** The two types biological functions of transcripts with higher expression.

**Seq**	**GO.ID**	**Term**	**Annotated**	**Significant**	**Expected**	**classicFisher**
1	GO:0043062	extracellular structure organization	445	26	3.9	1.80E−14
2	GO:0030198	extracellular matrix organization	384	24	3.37	4.70E−14
3	GO:0016477	cell migration	1537	40	13.47	3.00E−10
4	GO:0031012	extracellular matrix	519	29	4.48	1.10E−15
5	GO:0005578	proteinaceous extracellular matrix	410	26	3.54	1.90E−15
6	GO:0044420	extracellular matrix component	132	15	1.14	5.40E−13
7	GO:0005576	extracellular region	5443	89	46.94	1.30E−11
8	GO:0044421	extracellular region part	4578	75	39.48	1.90E−09
9	GO:0005615	extracellular space	4339	70	37.42	1.90E−08
10	GO:0070062	extracellular exosome	3105	52	26.77	1.00E−06
11	GO:1903561	extracellular vesicle	3127	52	26.96	1.20E−06
12	GO:0043230	extracellular organelle	3129	52	26.98	1.30E−06
13	GO:0005201	extracellular matrix structural	95	9	0.82	1.30E−07
14	GO:0050840	extracellular matrix binding	53	5	0.46	9.30E−05
1	GO:0022610	biological adhesion	1588	38	13.92	9.50E−09
2	GO:0007155	cell adhesion	1580	37	13.85	2.70E−08
3	GO:0030334	regulation of cell migration	917	26	8.04	1.20E−07
4	GO:0030155	regulation of cell adhesion	809	24	7.09	1.70E−07
5	GO:0031589	cell-substrate adhesion	350	14	3.07	2.70E−06
6	GO:0098609	cell–cell adhesion	955	24	8.37	3.30E−06
7	GO:0030154	cell differentiation	4541	65	39.8	1.00E−05
8	GO:0008283	cell proliferation	2299	40	20.15	1.50E−05
9	GO:0030335	positive regulation of cell migration	523	16	4.58	1.50E−05
10	GO:0048870	cell motility	1684	40	14.76	4.30E−09
11	GO:2000145	regulation of cell motility	970	26	8.5	3.60E−07
12	GO:2000147	positive regulation of cell motility	540	16	4.73	2.30E−05
13	GO:0050839	cell adhesion molecule binding	541	13	4.67	0.00087
14	GO:0005911	cell–cell junction	479	12	4.13	0.00095

By means of clusterProfiler analysis, a total of seven KEGG pathways from the KEGG database were found to be significantly enriched (Table 3). These enriched genes were not only significantly associated with focal adhesion (hsa04510, p_corrected_ = 0.00165) but also with the PI3K-Akt signaling pathway (hsa04151, p _corrected_ = 0.00087), which suggested that complex processes and mechanisms underlie the development of pterygium.

**Table 3:** The significantly enriched KEGG pathways of the up-regulation genes.

**KEGG ID**	**Description**	**Gene ratio**	**Bg ratio**	***P*-value**	**P.adjust**	***q*-value**	**Gene ID (enriched genes)**	**Count**
hsa04512	ECM-receptor interaction	8/88	82/7440	4.85E−06	0.0008	0.0007	3339∕3655∕3910∕7450∕1278∕1277∕22801∕2335	8
hsa04510	Focal adhesion	11/88	199/7440	2.01E−05	0.0016	0.0015	894∕3655∕3910∕857∕7450∕1278∕1277∕2318∕22801∕3479∕2335	11
hsa04974	Protein digestion and absorption	6/88	90/7440	0.00064825	0.0226	0.0207	1278∕1277∕2006∕7373∕1281∕1803	6
hsa05143	African trypanosomiasis	4/88	35/7440	0.000723215	0.0226	0.0207	3910∕3040∕3043∕3039	4
hsa04151	PI3K-Akt signaling pathway	12/88	354/7440	0.000871718	0.0226	0.0207	894∕3655∕3910∕7450∕1278∕1436∕1277∕2149∕22801∕3479∕2057∕2335	12
hsa04810	Regulation of actin cytoskeleton	9/88	213/7440	0.000877248	0.0226	0.0207	10163∕3655∕6387∕2149∕9459∕22801∕10788∕8395∕2335	9
hsa04640	Hematopoietic cell lineage	6/88	97/7440	0.000963238	0.0226	0.0207	3655∕947∕1436∕952∕1438∕2057	6

### Gene function analysis for the downregulated genes

GO analysis of the 139 downregulated genes revealed that these genes were mainly involved in inflammatory functions, including the “response to external stimulus” (GO:0009605, p_corrected_ = 0.000012), “antibacterial humoral response” (GO:0019731, p_corrected_ = 0.000017), “innate immune response in mucosa” (GO:0002227, p_corrected_ = 0.000028) and “NLRP1 inflammasome complex” (GO:0002227, p_corrected_ = 0.000028). Thus, the results suggest that inflammatory-related pathways are involved in the pathophysiology of pterygium. All enriched terms ranking in the top 50 based on the corrected *p*-values are listed in Table S2. We did not find any KEGG pathways from the KEGG database that were significantly enriched because of the limited number of input genes.

### WGCNA

**Figure 2:**
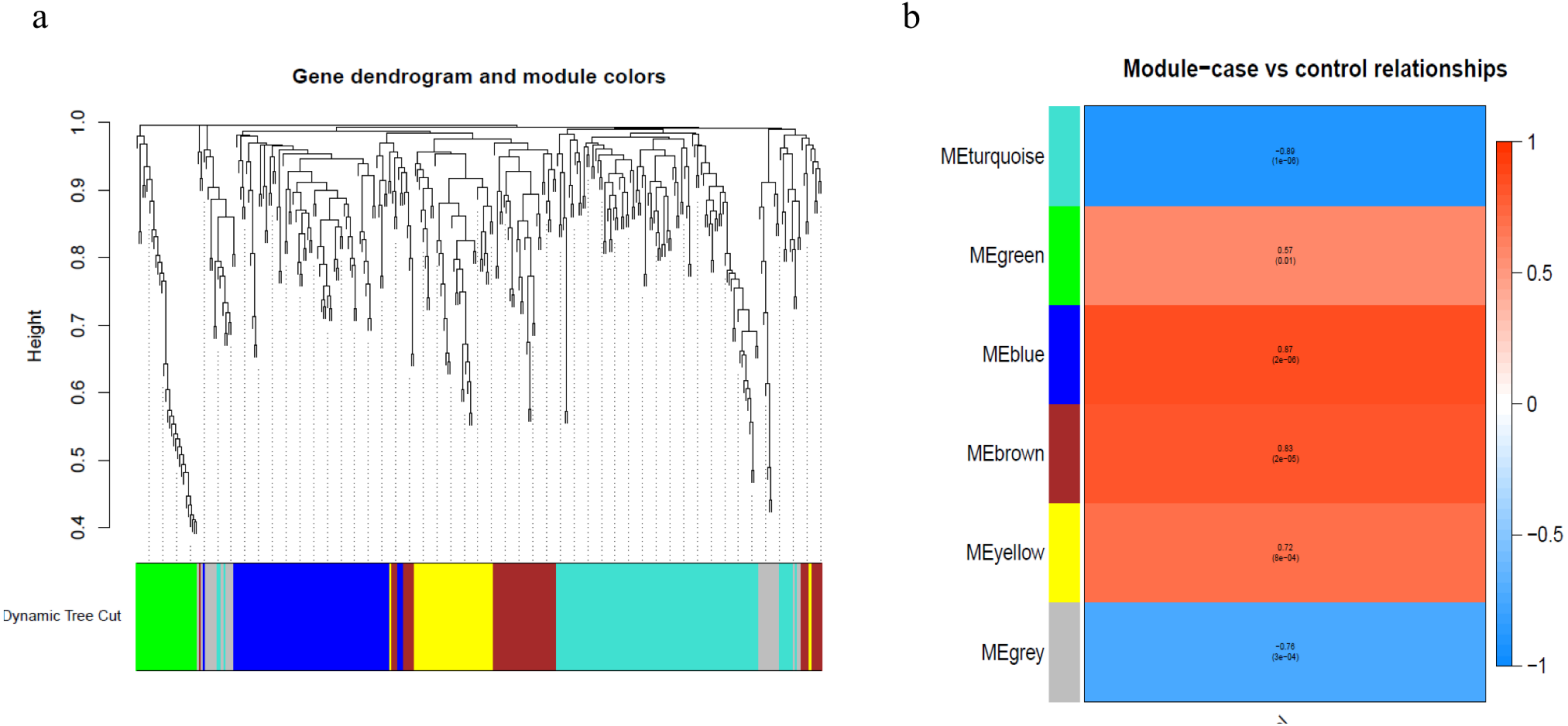
Module assignment dendrogram plot and association between module and trait in the WGCNA. (A) The gene dendrogram and module assignment for the total DEGs. (B) The correlation analysis between five modules (exclude the gray) and phenotypic comparisons between pterygium and normal conjunctival tissues.

**Figure 3:**
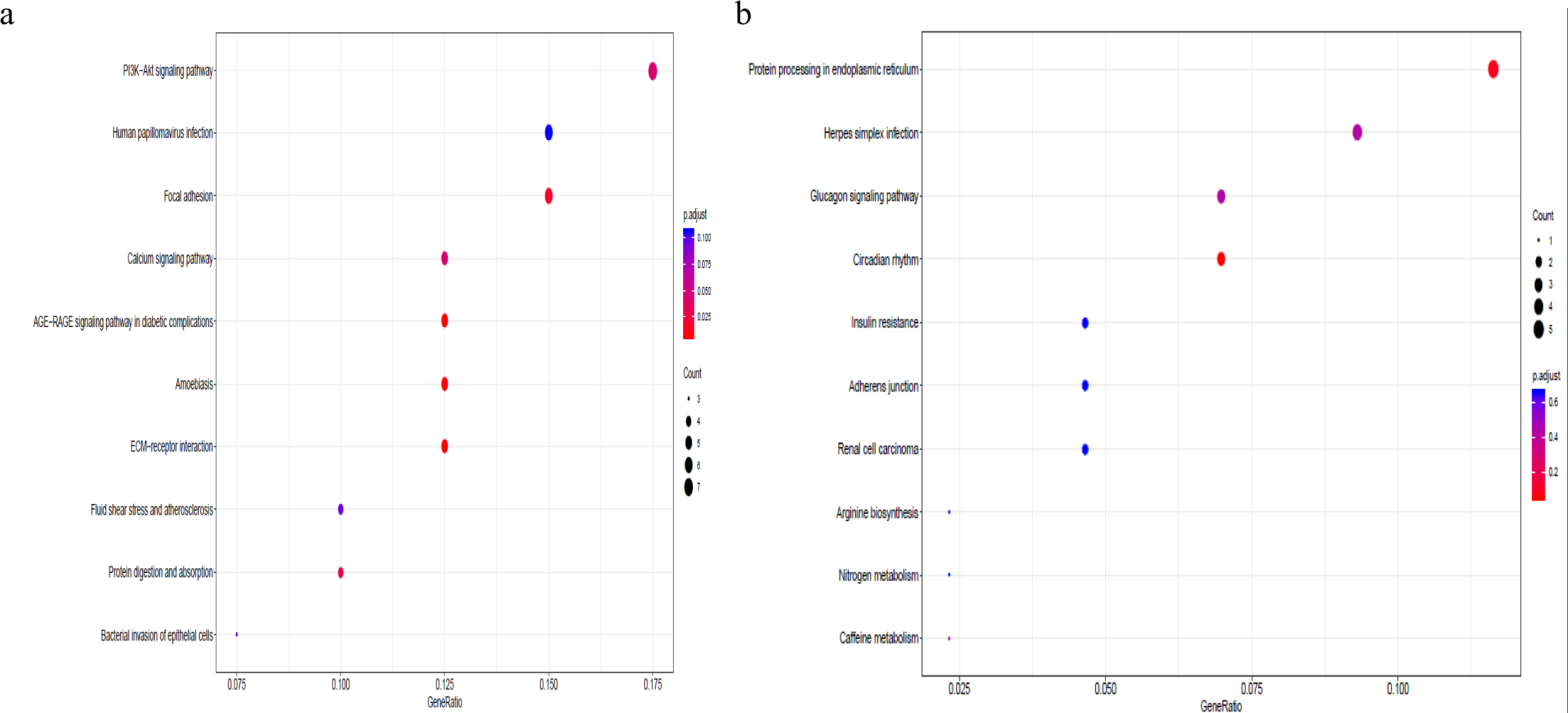
Gene set pathway enrichment analysis. (A) The enriched pathways in the blue module and PI3K-Akt pathway was of top significance; (B) the biological pathways in the turquoise module and endoplasmic reticulum related pathway were the most significant association.

**Figure 4:**
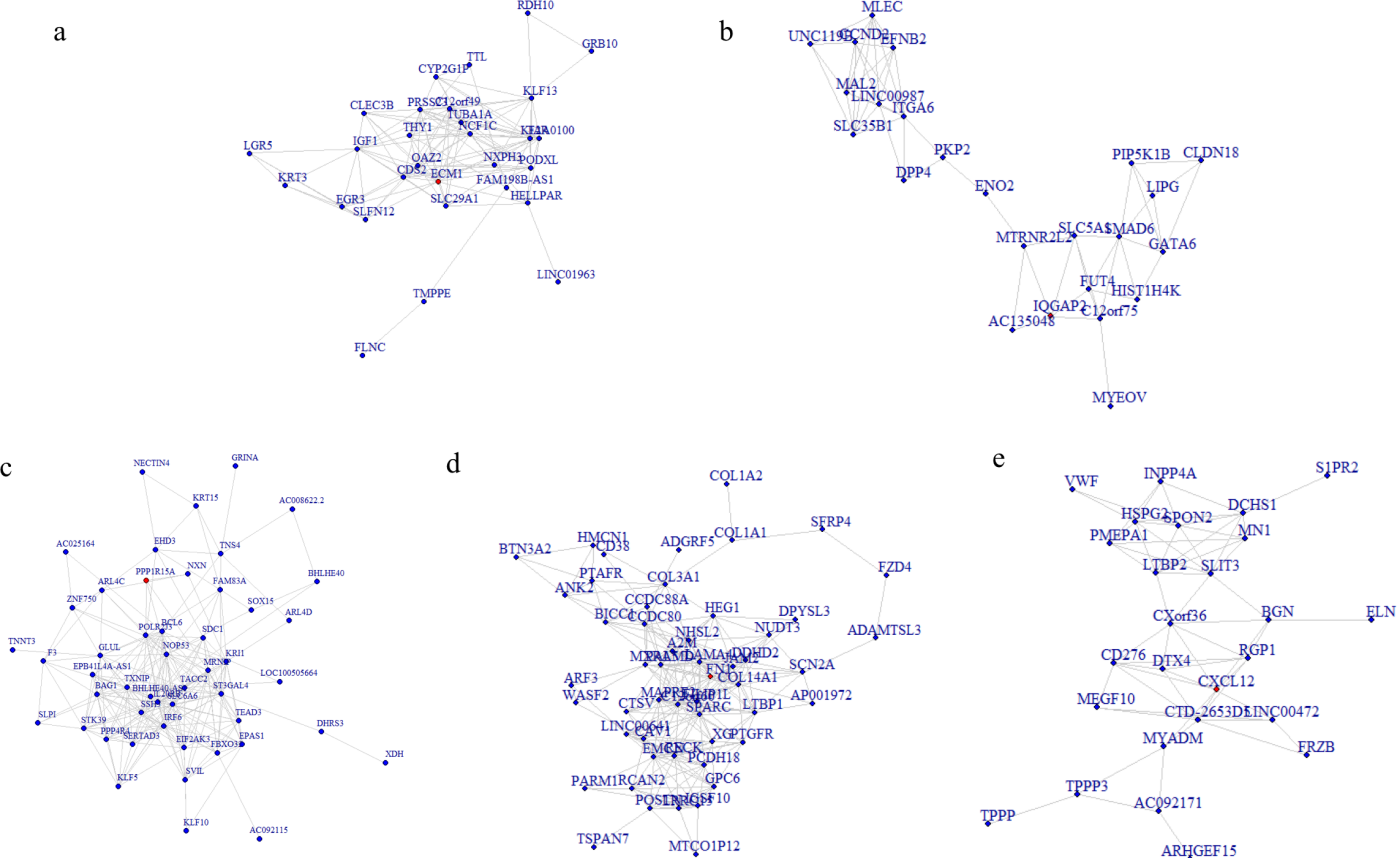
Network analysis for the five modules and hub gene represented as the red dot in each network plot. (A) Network plot for the yellow module and ECM1 as the hub gene in the expressed network; (B) IQGAP2 as the hub gene in the green module; (C) GADD34 gene in the turquoise module; (D) FN1 gene in the blue module; (E) CXCL12 in the brown module.

To better understand the co-expression patterns of the DEGs, we performed WGCNA using the counts of each gene in the individual samples. First, the soft-thresholding power was calculated to be equal to 10 with the pickSoftThreshold program. By means of the dynamicTreeCut function, five modules were detected using the topological overlap matrix and corresponding dissimilarity approach; the module assignments are shown under the gene dendrogram in Fig. 2A. Then, we combined the module relationships and clinical traits and quantified the module trait associations. The results revealed that the turquoise module was negatively associated with the phenotypic comparison, while the other four modules were positively correlated with the phenotypic comparison (Fig. 2B). Interestingly, the PI3K-Akt pathway was the most significantly enriched pathway in the blue module, which was positively associated with pterygium, while the protein processing in the endoplasmic reticulum (ER) pathway was enriched in the turquoise module, which was negatively associated with pterygium (Fig. 3). We speculate that pterygium is associated with ER stress-related biological activity. We further annotated the biological functions for each module, and the top 50 biological process (BP), cellular component (CC) and molecular function (MF) terms are listed in Table S3. Next, the package TopHubInEachModule was used to provide an easy way to identify the hub gene from every module. Five hub genes were identified from the five modules. These five hub genes were ECM1 in the yellow module (kME = 0.97), IQGAP2 in the green module (kME = 0.969), PPP1R15A (GADD34) in the turquoise module (kME = 0.939), FN1 in the blue module (kME = 0.995) and CXCL12 in the brown module (kME = 0.965). To demonstrate the connectivity of the five hub genes in the network, we plotted the network using the adjacency matrix of the eigengenes in each module and highlighted the hub genes in the network (Fig. 4). By means of the PEEP method, the set of genes which were significantly perturbed in each single subject were shown in Table S4 , which had potential for diagnosis and treatment of pterygium.

### Validation of mRNA expression changes in the top DEGs and hub genes in the pterygium

**Figure 5:**
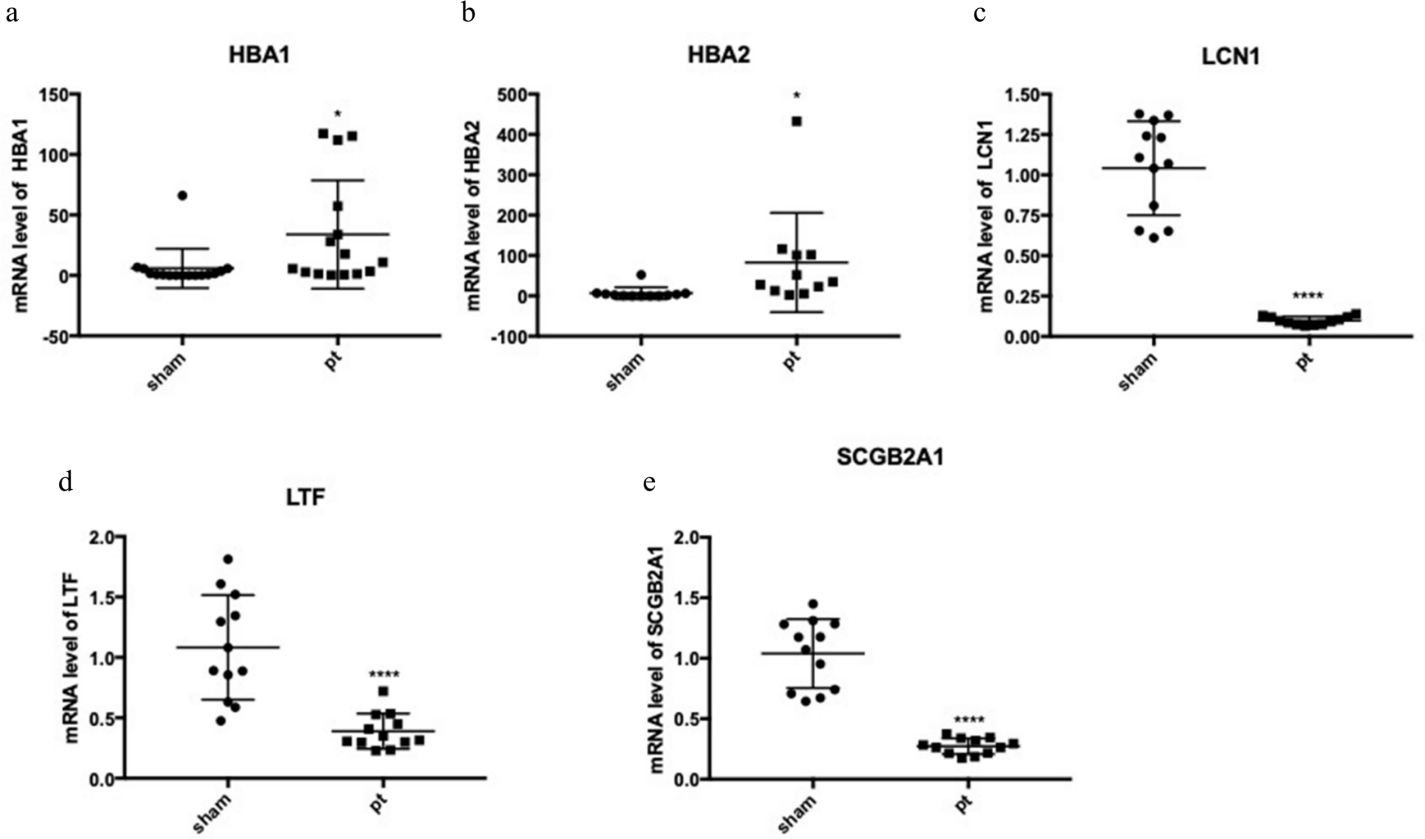
qRT-PCR to detect the expression of the top differentially expressed genes (DEGs). (A) HBA1; (B) HBA2; (C) LCN1; (D) LTF; (E) SCGB2A1.

**Figure 6:**
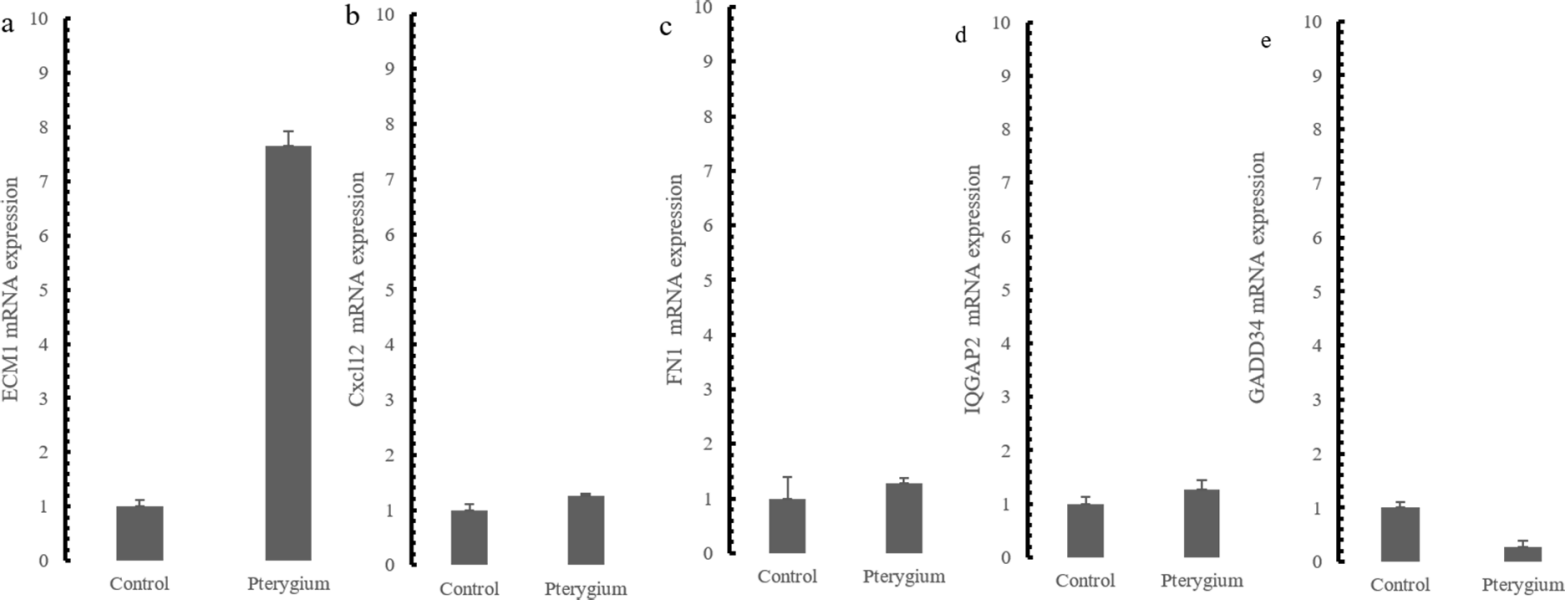
qRT-PCR Validation for hub genes mRNA level in the independent samples. (A) ECM1 up-regulated expression in the comparison between pterygium and control; (B) CXCL12 mRNA expression; (C) FN1 mRNA expression; (D) IQGAP2 mRNA expression (E) GADD34 mRNA expression.

The expression of the five hub genes (ECM1, IQGAP2, FN1, GADD34, CXCL12) was determined by real-time PCR in comparisons between 10 normal conjunctival tissues and 10 pterygium tissues. The housekeeping gene GAPDH was not significantly altered between the two groups (*p* = 0.697) and was used to normalize the expression of the five genes. From the results of RNA-Seq, LCN1, LTF, SCGB2A1, HBA1 (hemoglobin subunit alpha 1) and HBA2 (hemoglobin subunit alpha 2) ranked as the top five DEGs in the comparison between pterygium and normal tissues. We successfully validated the results of RNA-Seq in an independent sample cohort. The mRNA expressions of SCGB2A1, LTF and LCN1 were significantly decreased in pterygium tissues compared with normal control tissues, while the mRNA levels of HBA1 and HBA2 were higher in the pterygium tissues than in the control tissues (Fig. 5). As represented in Fig. 6, ECM1 gene expression significantly increased in the pterygium group compared with the control group (7.64-fold increase, *p* = 0.0036). The other three genes, FN1, CXCL12 and IQGAP2, have higher mRNA levels, although the differences were statistically insignificant (FN1: 1.78-fold increase, *p* = 0.155; CXCL12: 1.25-fold increase, *p* = 0.24; IQGAP2: 1.274-fold increase, *p* = 0.283). The expression of GADD34, on the other hand, was notably decreased in the pterygium group (0.277-fold decrease, *p* = 0.005).

## Discussion

Earlier, several groups have conducted DNA microarray studies on pterygium; however, few studies have used RNA-Seq, which has advantages over DNA microarray analysis (Rai et al., 2018). Bang et al. (2017) utilized RNA-Seq to explore complement factors and found that several factors were dysregulated in pterygium compared to normal conjunctival tissues (Bang et al., 2017). Enlightened by the previous research on gene expression of pterygium, one main feature of this study is to take the lead to integrate RNA-Seq and bioinformatic analysis methods to explore whole genome transcription in pterygium tissues and is the first study attempting to use the WGCNA method to explore the mechanisms of the disease.

### Links to cell adhesion and extracellular matrix remodeling

Since pterygium is an ocular surface disease featuring excessive vascular ingrowth and the accumulation of extracellular matrix, the abnormal expression of extracellular matrix proteins could be related to the formation of pterygium.

According to the results of WGCNA performed on the DEGs, we identified five hub genes in five modules, including ECM1, IQGAP2, GADD34, FN1 and CXCL12. Confirmed by qRT-PCR validation, three out of the five genes were associated with cell adhesion and extracellular matrix remodeling. This is consistent with a previous study showing that ECM1, which encodes extracellular matrix protein 1, was found significantly increased in pterygium. ECM1 showed the highest similarity in the yellow module. Earlier, John-Aryankalayil et al. (2006) reported that upregulation of the ECM1 gene plays a key role in pterygium, as has also been validated by several other studies (Naib-Majani et al., 2004; Turner et al., 2007). FN1 encodes for fibronectin 1, which is a crucial glycoprotein in cell adhesion and migration during embryogenesis, wound healing and metastasis; and higher expression of FN1 has been observed in pterygium tissues (Engelsvold et al., 2013b). A previous study examined significant alterations in FN1 through DNA microarray analysis and reported that FN1 serves as a potential regulator of epithelial cell migration, extracellular matrix deposition and the epithelial-mesenchymal transition in pterygium (Engelsvold et al., 2013a). IQGAP2 is a signal-transducing scaffold protein that acts as an integrator of Rho GTPase and Ca^2+^/calmodulin signals associated with cell adhesion and cytoskeletal reorganization. A study also reported that IQGAP2 plays a role in regulating Wnt/ β-catenin and PI3K/Akt signaling (Schmidt et al., 2003). Interestingly, intranuclear accumulation of β-catenin in pterygium tissues has been reported (Kato et al., 2007). However, the functions of IQGAP2 in pterygium have not been addressed. Our study may provide new insights into the role of IQGAP2 in the mechanisms of pterygium pathogenesis.

### Links to immunology

Several potential pathogenesis mechanisms for the ingrowth of pterygium have been reported dating back to last century, including immunological mechanisms (Pinkerton, Hokama & Shigemura, 1984) and increased cell stress (Kau et al., 2006). The results of WGCNA showed that CXCL12 and GADD34 were the hub genes in brown module and turquoise modules, respectively. CXCL12 (also called SDF-1) encodes for stromal cell-derived factor-1, an angiogenic chemokine. It plays a role in diverse cellular functions, including embryogenesis, immune surveillance, inflammation responses and so on. CXCL1 promotes angiogenesis through CXCR2 (Miyake et al., 2013) and regulates the recruitment of granulocytes during the inflammatory process (Geiser et al., 1993). Bamdad et al. (2017) reported that upregulation of SDF-1 contributes to pterygium (Bamdad et al., 2017). Kim et al. (2013) reported that the levels of CXCL12 and CXCR4 can be used to determine the severity of pterygium (Kim et al., 2013). GADD34 is a growth cycle protein that could be induced by growth arrest, DNA damage, and other kinds of cell stress. When intracellular proteins cannot fold properly, the disruption of endoplasmic reticulum (ER) physiological function leads to ‘endoplasmic reticulum stress’. A long period of ER stress can induce the expression of GADD34 (Reid et al., 2016). Correspondingly, we also found that the ER stress-related pathway plays a contributory role in the development of pterygium according to the RNA-Seq results.

On the basis of RNA-Seq, the top low expressed DEGs were LCN1, LTF and SCGB2A1 (LCN1: 0.094-fold decrease, *p* < 0.0001; LTF: 0.36-fold decrease, *p* < 0.0001; SCGB2A1: 0.26-fold decrease, *p* < 0.0001) , which were associated with immune process. LCN1, encodes a member of the lipocalin family, is believed to be involved in innate immune response. The expression of LCN1 was found to be significantly increased in breast cancer tissues compared with adjacent normal tissues, possibly resulting from more neoantigens in cancer patients and therefore more immune infiltration (Yang et al., 2019). LTF is an important component of the innate immune system. The other three genes have not been reported in previous pterygium studies. However, the protein levels of these three downregulated genes have been reported to be perturbed in dry eye syndrome patients (Perumal et al., 2016). Dry eye is an important risk factor for the formation of primary or recurrent pterygium, and pterygium is also associated with ocular surface instability and dry eye disease (Ozsutcu et al., 2014). Many studies have attempted to explain the relationship between pterygium and dry eye. Based on our study, attention should be paid to genes such as LCN1, LTF and SCGB2A1, which had lower expression levels in pterygium, to determine whether they are directly involved in the development of pterygium and dry eye.

In conclusion, in this study, we strictly selected subjects and matched the demographic information, including age, gender, etc., between the case and control group. A range of genes and proteins were found to be aberrantly expressed in pterygium tissues, including growth factors, matrix metalloproteinases, interleukins, proliferation-related proteins, apoptosis-related proteins, cell adhesion molecules, tight junction proteins and endoplasmic reticulum stress response pathway-related molecules. The current study also confirmed the roles of key biological activities that have been reported in studies on the molecular mechanisms of pterygium.

All in all, this study has mined deeper into the pterygium transcriptome by applying a combination of RNA-Seq and bioinformatic analysis methods for the first time. We speculate that the genes identified to be associated with pterygium mutually interact and form a complex molecular network. It is possible that the significant dysregulation of the hub genes directly perturbs the entire network, contributing to the initiation and development of pterygium. Thus, pharmaceutical interventions targeting these hub genes might be effective for the treatment of pterygium.

There were some limitations to our study. First, the sample size was relatively small, and we also did not classify pterygium based on morphologic and pathologic characteristics. Second, we did not conduct an in vitro cell experiment to verify the potential mechanism of the ER stress-related pathway. Third, the lack of functional validation of certain hub genes requires future research. Last but not least, many important risk factors, such as ultraviolet irradiation or chronic ocular inflammation could directly affect the development of pterygium. Due to the limited information on this, we did not systematically analyze the correlation between risk factors and the transcriptional data.

## Conclusion

Taken together, our RNA-Seq data confirm the dysregulated genes that have been published in a DNA microarray study. In addition, inflammatory, cell adhesion and extracellular matrix pathways were found to be enriched for DEGs in pterygium. By WGCNA, we identified five key hub genes and a novel biological pathway involved in the development of pterygium.

##  Supplemental Information

10.7717/peerj.9056/supp-1 Table S1 The top 50 GO terms including BP, CC and MP for the up-regulated genes

10.7717/peerj.9056/supp-2 Table S2 The top 50 GO terms including BP, CC and MP for the down-regulated genes

10.7717/peerj.9056/supp-3 Table S3 Gene Ontology of each module in the WGCNA analysis

10.7717/peerj.9056/supp-4 Table S4 Genes significantly perturbed in each single subject with PEEP method

10.7717/peerj.9056/supp-5 Supplemental Information 1 Raw data for Fig. 5

10.7717/peerj.9056/supp-6 Supplemental Information 6 Raw data for Fig. 6

## Additional Information and Declarations

### Data Availability

The following information was supplied regarding data availability: All data are available at the NODE database: OEP000653.
