# Supplementary material for: Transcriptional profiling to identify the key genes and pathways of pterygium
Source: PeerJ. 2020 May 4;8:e9056. doi: 10.7717/peerj.9056 (PMC7204871; doi:10.7717/peerj.9056)
Supplement: Table S1 [file peerj-08-9056-s001.docx]

**Supplementary table 1 The top 50 GO terms including BP, CC and MP for the up-regulated genes**

| **Function** | **Rank** | **GO.ID** | **Term** | **Annotated** | **Significant** | **Expected** | **classicFisher** |
| --- | --- | --- | --- | --- | --- | --- | --- |
| BP | 1 | GO:0043062 | extracellular structure organization | 445 | 26 | 3.9 | 1.80E-14 |
|  | 2 | GO:0030198 | extracellular matrix organization | 384 | 24 | 3.37 | 4.70E-14 |
|  | 3 | GO:0016477 | cell migration | 1537 | 40 | 13.47 | 3.00E-10 |
|  | 4 | GO:0006928 | movement of cell or subcellular componen... | 2185 | 48 | 19.15 | 1.00E-09 |
|  | 5 | GO:0072359 | circulatory system development | 1155 | 33 | 10.12 | 1.50E-09 |
|  | 6 | GO:0040011 | locomotion | 1955 | 44 | 17.14 | 3.10E-09 |
|  | 7 | GO:0048870 | cell motility | 1684 | 40 | 14.76 | 4.30E-09 |
|  | 8 | GO:0051674 | localization of cell | 1684 | 40 | 14.76 | 4.30E-09 |
|  | 9 | GO:0032502 | developmental process | 6840 | 97 | 59.95 | 5.60E-09 |
|  | 10 | GO:0001944 | vasculature development | 790 | 26 | 6.92 | 6.10E-09 |
|  | 11 | GO:0051239 | regulation of multicellular organismal p... | 3303 | 60 | 28.95 | 6.90E-09 |
|  | 12 | GO:0072358 | cardiovascular system development | 801 | 26 | 7.02 | 8.10E-09 |
|  | 13 | GO:0048856 | anatomical structure development | 6364 | 92 | 55.78 | 8.50E-09 |
|  | 14 | GO:0009653 | anatomical structure morphogenesis | 2903 | 55 | 25.45 | 9.20E-09 |
|  | 15 | GO:0022610 | biological adhesion | 1588 | 38 | 13.92 | 9.50E-09 |
|  | 16 | GO:0048731 | system development | 5191 | 80 | 45.5 | 1.10E-08 |
|  | 17 | GO:0001568 | blood vessel development | 765 | 25 | 6.71 | 1.40E-08 |
|  | 18 | GO:0007275 | multicellular organism development | 5844 | 86 | 51.22 | 1.90E-08 |
|  | 19 | GO:0007155 | cell adhesion | 1580 | 37 | 13.85 | 2.70E-08 |
|  | 20 | GO:0051270 | regulation of cellular component movemen... | 1056 | 29 | 9.26 | 4.10E-08 |
|  | 21 | GO:0032501 | multicellular organismal process | 8327 | 108 | 72.99 | 5.40E-08 |
|  | 22 | GO:0030334 | regulation of cell migration | 917 | 26 | 8.04 | 1.20E-07 |
|  | 23 | GO:0030155 | regulation of cell adhesion | 809 | 24 | 7.09 | 1.70E-07 |
|  | 24 | GO:0071559 | response to transforming growth factor b... | 246 | 13 | 2.16 | 2.70E-07 |
|  | 25 | GO:2000145 | regulation of cell motility | 970 | 26 | 8.5 | 3.60E-07 |
|  | 26 | GO:0050793 | regulation of developmental process | 2807 | 50 | 24.6 | 4.00E-07 |
|  | 27 | GO:2000026 | regulation of multicellular organismal d... | 2173 | 42 | 19.05 | 5.60E-07 |
|  | 28 | GO:0040012 | regulation of locomotion | 1076 | 27 | 9.43 | 7.50E-07 |
|  | 29 | GO:0071560 | cellular response to transforming growth... | 242 | 12 | 2.12 | 1.60E-06 |
|  | 30 | GO:0007166 | cell surface receptor signaling pathway | 3382 | 55 | 29.64 | 1.60E-06 |
|  | 31 | GO:0035295 | tube development | 1141 | 27 | 10 | 2.30E-06 |
|  | 32 | GO:0048513 | animal organ development | 3789 | 59 | 33.21 | 2.40E-06 |
|  | 33 | GO:0031589 | cell-substrate adhesion | 350 | 14 | 3.07 | 2.70E-06 |
|  | 34 | GO:0035239 | tube morphogenesis | 947 | 24 | 8.3 | 2.80E-06 |
|  | 35 | GO:0098609 | cell-cell adhesion | 955 | 24 | 8.37 | 3.30E-06 |
|  | 36 | GO:0010524 | positive regulation of calcium ion trans... | 52 | 6 | 0.46 | 6.10E-06 |
|  | 37 | GO:0035904 | aorta development | 52 | 6 | 0.46 | 6.10E-06 |
|  | 38 | GO:0003013 | circulatory system process | 548 | 17 | 4.8 | 6.90E-06 |
|  | 39 | GO:0051928 | positive regulation of calcium ion trans... | 113 | 8 | 0.99 | 6.90E-06 |
|  | 40 | GO:0048514 | blood vessel morphogenesis | 682 | 19 | 5.98 | 9.00E-06 |
|  | 41 | GO:0030154 | cell differentiation | 4541 | 65 | 39.8 | 1.00E-05 |
|  | 42 | GO:0048869 | cellular developmental process | 4750 | 67 | 41.63 | 1.20E-05 |
|  | 43 | GO:0008283 | cell proliferation | 2299 | 40 | 20.15 | 1.50E-05 |
|  | 44 | GO:0030335 | positive regulation of cell migration | 523 | 16 | 4.58 | 1.50E-05 |
|  | 45 | GO:0023052 | signaling | 7642 | 94 | 66.98 | 2.00E-05 |
|  | 46 | GO:1904427 | positive regulation of calcium ion trans... | 65 | 6 | 0.57 | 2.20E-05 |
|  | 47 | GO:2000147 | positive regulation of cell motility | 540 | 16 | 4.73 | 2.30E-05 |
|  | 48 | GO:0065008 | regulation of biological quality | 4163 | 60 | 36.49 | 2.30E-05 |
|  | 49 | GO:0070848 | response to growth factor | 731 | 19 | 6.41 | 2.40E-05 |
|  | 50 | GO:0010522 | regulation of calcium ion transport into... | 98 | 7 | 0.86 | 2.50E-05 |
| CC | 1 | GO:0031012 | extracellular matrix | 519 | 29 | 4.48 | 1.10E-15 |
|  | 2 | GO:0031720 | haptoglobin binding | 3 | 3 | 0.03 | 6.30E-07 |
|  | 3 | GO:0031012 | extracellular matrix | 519 | 29 | 4.48 | 1.10E-15 |
|  | 4 | GO:0005578 | proteinaceous extracellular matrix | 410 | 26 | 3.54 | 1.90E-15 |
|  | 5 | GO:0044420 | extracellular matrix component | 132 | 15 | 1.14 | 5.40E-13 |
|  | 6 | GO:0005576 | extracellular region | 5443 | 89 | 46.94 | 1.30E-11 |
|  | 7 | GO:0044421 | extracellular region part | 4578 | 75 | 39.48 | 1.90E-09 |
|  | 8 | GO:0005615 | extracellular space | 4339 | 70 | 37.42 | 1.90E-08 |
|  | 9 | GO:0031982 | vesicle | 4920 | 73 | 42.43 | 2.70E-07 |
|  | 10 | GO:0070062 | extracellular exosome | 3105 | 52 | 26.77 | 1.00E-06 |
|  | 11 | GO:1903561 | extracellular vesicle | 3127 | 52 | 26.96 | 1.20E-06 |
|  | 12 | GO:0043230 | extracellular organelle | 3129 | 52 | 26.98 | 1.30E-06 |
|  | 13 | GO:0005604 | basement membrane | 92 | 8 | 0.79 | 1.30E-06 |
|  | 14 | GO:0009986 | cell surface | 936 | 24 | 8.07 | 1.80E-06 |
|  | 15 | GO:0031838 | haptoglobin-hemoglobin complex | 4 | 3 | 0.03 | 2.50E-06 |
|  | 16 | GO:0071682 | endocytic vesicle lumen | 20 | 4 | 0.17 | 2.30E-05 |
|  | 17 | GO:0060205 | cytoplasmic vesicle lumen | 392 | 13 | 3.38 | 3.80E-05 |
|  | 18 | GO:0031983 | vesicle lumen | 393 | 13 | 3.39 | 3.90E-05 |
|  | 19 | GO:0031093 | platelet alpha granule lumen | 74 | 6 | 0.64 | 4.30E-05 |
|  | 20 | GO:0031410 | cytoplasmic vesicle | 2636 | 42 | 22.73 | 5.10E-05 |
|  | 21 | GO:0045177 | apical part of cell | 404 | 13 | 3.48 | 5.10E-05 |
|  | 22 | GO:0097708 | intracellular vesicle | 2639 | 42 | 22.76 | 5.20E-05 |
|  | 23 | GO:0009897 | external side of plasma membrane | 295 | 11 | 2.54 | 5.30E-05 |
|  | 24 | GO:0005584 | collagen type I trimer | 2 | 2 | 0.02 | 7.40E-05 |
|  | 25 | GO:0030141 | secretory granule | 959 | 21 | 8.27 | 8.40E-05 |
|  | 26 | GO:0099512 | supramolecular fiber | 1046 | 22 | 9.02 | 0.0001 |
|  | 27 | GO:0099081 | supramolecular polymer | 1053 | 22 | 9.08 | 0.00011 |
|  | 28 | GO:0099080 | supramolecular complex | 1054 | 22 | 9.09 | 0.00011 |
|  | 29 | GO:0005833 | hemoglobin complex | 12 | 3 | 0.1 | 0.00013 |
|  | 30 | GO:0045121 | membrane raft | 336 | 11 | 2.9 | 0.00017 |
|  | 31 | GO:0098857 | membrane microdomain | 337 | 11 | 2.91 | 0.00017 |
|  | 32 | GO:0099503 | secretory vesicle | 1106 | 22 | 9.54 | 0.00022 |
|  | 33 | GO:0098589 | membrane region | 348 | 11 | 3 | 0.00023 |
|  | 34 | GO:0031091 | platelet alpha granule | 103 | 6 | 0.89 | 0.00027 |
|  | 35 | GO:0012505 | endomembrane system | 5028 | 64 | 43.36 | 0.00034 |
|  | 36 | GO:0044459 | plasma membrane part | 3442 | 48 | 29.68 | 0.00035 |
|  | 37 | GO:0030027 | lamellipodium | 204 | 8 | 1.76 | 0.0004 |
|  | 38 | GO:0005911 | cell-cell junction | 479 | 12 | 4.13 | 0.00095 |
|  | 39 | GO:0005887 | integral component of plasma membrane | 2106 | 32 | 18.16 | 0.00107 |
|  | 40 | GO:0005583 | fibrillar collagen trimer | 25 | 3 | 0.22 | 0.00126 |
|  | 41 | GO:0098643 | banded collagen fibril | 25 | 3 | 0.22 | 0.00126 |
|  | 42 | GO:0034774 | secretory granule lumen | 374 | 10 | 3.23 | 0.00156 |
|  | 43 | GO:0031226 | intrinsic component of plasma membrane | 2174 | 32 | 18.75 | 0.0018 |
|  | 44 | GO:0071944 | cell periphery | 6532 | 75 | 56.33 | 0.00201 |
|  | 45 | GO:0072562 | blood microparticle | 152 | 6 | 1.31 | 0.00208 |
|  | 46 | GO:0016324 | apical plasma membrane | 335 | 9 | 2.89 | 0.00258 |
|  | 47 | GO:0098644 | complex of collagen trimers | 33 | 3 | 0.28 | 0.00284 |
|  | 48 | GO:0043202 | lysosomal lumen | 112 | 5 | 0.97 | 0.00288 |
|  | 49 | GO:0031252 | cell leading edge | 411 | 10 | 3.54 | 0.00309 |
|  | 50 | GO:0001527 | microfibril | 10 | 2 | 0.09 | 0.00318 |
| MF | 1 | GO:0005886 | plasma membrane | 6420 | 73 | 55.36 | 0.00319 |
|  | 2 | GO:0002116 | semaphorin receptor complex | 11 | 2 | 0.09 | 0.00386 |
|  | 3 | GO:0005201 | extracellular matrix structural constitu... | 95 | 9 | 0.82 | 1.30E-07 |
|  | 4 | GO:0031720 | haptoglobin binding | 3 | 3 | 0.03 | 6.30E-07 |
|  | 5 | GO:0005178 | integrin binding | 129 | 9 | 1.11 | 1.80E-06 |
|  | 6 | GO:0019838 | growth factor binding | 145 | 9 | 1.25 | 4.80E-06 |
|  | 7 | GO:0005518 | collagen binding | 79 | 6 | 0.68 | 6.30E-05 |
|  | 8 | GO:0050840 | extracellular matrix binding | 53 | 5 | 0.46 | 9.30E-05 |
|  | 9 | GO:1901681 | sulfur compound binding | 262 | 10 | 2.26 | 9.70E-05 |
|  | 10 | GO:0048407 | platelet-derived growth factor binding | 11 | 3 | 0.1 | 9.90E-05 |
|  | 11 | GO:0002020 | protease binding | 169 | 8 | 1.46 | 0.00011 |
|  | 12 | GO:0005102 | signaling receptor binding | 1810 | 31 | 15.64 | 0.00017 |
|  | 13 | GO:0005344 | oxygen carrier activity | 14 | 3 | 0.12 | 0.00022 |
|  | 14 | GO:0005539 | glycosaminoglycan binding | 240 | 9 | 2.07 | 0.00025 |
|  | 15 | GO:0005198 | structural molecule activity | 809 | 17 | 6.99 | 0.00066 |
|  | 16 | GO:0050839 | cell adhesion molecule binding | 541 | 13 | 4.67 | 0.00087 |
|  | 17 | GO:0042813 | Wnt-activated receptor activity | 23 | 3 | 0.2 | 0.00099 |
|  | 18 | GO:0008201 | heparin binding | 184 | 7 | 1.59 | 0.00111 |
|  | 19 | GO:0043236 | laminin binding | 29 | 3 | 0.25 | 0.00196 |
|  | 20 | GO:0044877 | protein-containing complex binding | 1071 | 19 | 9.25 | 0.00232 |
|  | 21 | GO:0017147 | Wnt-protein binding | 32 | 3 | 0.28 | 0.00261 |
|  | 22 | GO:0008238 | exopeptidase activity | 111 | 5 | 0.96 | 0.00278 |
|  | 23 | GO:0019955 | cytokine binding | 116 | 5 | 1 | 0.00337 |
|  | 24 | GO:0016175 | superoxide-generating NADPH oxidase acti... | 11 | 2 | 0.1 | 0.00388 |
|  | 25 | GO:0017154 | semaphorin receptor activity | 11 | 2 | 0.1 | 0.00388 |
|  | 26 | GO:0046332 | SMAD binding | 77 | 4 | 0.67 | 0.00446 |
|  | 27 | GO:0008239 | dipeptidyl-peptidase activity | 12 | 2 | 0.1 | 0.00463 |
|  | 28 | GO:0030674 | protein binding, bridging | 185 | 6 | 1.6 | 0.00548 |
|  | 29 | GO:0005509 | calcium ion binding | 752 | 14 | 6.5 | 0.00578 |
|  | 30 | GO:0140104 | molecular carrier activity | 43 | 3 | 0.37 | 0.00607 |
|  | 31 | GO:0004180 | carboxypeptidase activity | 45 | 3 | 0.39 | 0.00689 |
|  | 32 | GO:0004177 | aminopeptidase activity | 46 | 3 | 0.4 | 0.00733 |
|  | 33 | GO:0050664 | oxidoreductase activity, acting on NAD(P... | 16 | 2 | 0.14 | 0.00823 |
|  | 34 | GO:0019901 | protein kinase binding | 708 | 13 | 6.12 | 0.00862 |
|  | 35 | GO:0004835 | tubulin-tyrosine ligase activity | 1 | 1 | 0.01 | 0.00864 |
|  | 36 | GO:0004900 | erythropoietin receptor activity | 1 | 1 | 0.01 | 0.00864 |
|  | 37 | GO:0033142 | progesterone receptor binding | 1 | 1 | 0.01 | 0.00864 |
|  | 38 | GO:0036143 | kringle domain binding | 1 | 1 | 0.01 | 0.00864 |
|  | 39 | GO:0070320 | inward rectifier potassium channel inhib... | 1 | 1 | 0.01 | 0.00864 |
|  | 40 | GO:0004601 | peroxidase activity | 49 | 3 | 0.42 | 0.00873 |
|  | 41 | GO:0008092 | cytoskeletal protein binding | 960 | 16 | 8.3 | 0.0091 |
|  | 42 | GO:0060090 | molecular adaptor activity | 206 | 6 | 1.78 | 0.0091 |
|  | 43 | GO:0019899 | enzyme binding | 2527 | 33 | 21.83 | 0.00956 |
|  | 44 | GO:0019825 | oxygen binding | 51 | 3 | 0.44 | 0.00974 |
|  | 45 | GO:0008047 | enzyme activator activity | 568 | 11 | 4.91 | 0.01055 |
|  | 46 | GO:0016684 | oxidoreductase activity, acting on perox... | 54 | 3 | 0.47 | 0.01139 |
|  | 47 | GO:0008017 | microtubule binding | 283 | 7 | 2.45 | 0.01168 |
|  | 48 | GO:0030234 | enzyme regulator activity | 1167 | 18 | 10.08 | 0.01228 |
|  | 49 | GO:0005001 | transmembrane receptor protein tyrosine ... | 20 | 2 | 0.17 | 0.01273 |
|  | 50 | GO:0019198 | transmembrane receptor protein phosphata... | 20 | 2 | 0.17 | 0.01273 |
