# Supplementary material for: Transcriptional profiling to identify the key genes and pathways of pterygium
Source: PeerJ. 2020 May 4;8:e9056. doi: 10.7717/peerj.9056 (PMC7204871; doi:10.7717/peerj.9056)
Supplement: Table S2 [file peerj-08-9056-s002.docx]

**Supplementary table2 The top 50 GO terms including BP, CC and MP for the down-regulated genes**

| **Function** | **Rank** | **GO.ID** | **Term** | **Annotated** | **Significant** | **Expected** | **Classic Fisher** |
| --- | --- | --- | --- | --- | --- | --- | --- |
| BP | 1 | GO:0000122 | negative regulation of transcription by ... | 863 | 21 | 4.93 | 1.60E-08 |
|  | 2 | GO:0051172 | negative regulation of nitrogen compound... | 2839 | 38 | 16.2 | 1.80E-07 |
|  | 3 | GO:0045892 | negative regulation of transcription, DN... | 1283 | 24 | 7.32 | 2.00E-07 |
|  | 4 | GO:0031324 | negative regulation of cellular metaboli... | 3003 | 39 | 17.14 | 2.50E-07 |
|  | 5 | GO:1903507 | negative regulation of nucleic acid-temp... | 1331 | 24 | 7.6 | 3.80E-07 |
|  | 6 | GO:1902679 | negative regulation of RNA biosynthetic ... | 1333 | 24 | 7.61 | 3.90E-07 |
|  | 7 | GO:0009890 | negative regulation of biosynthetic proc... | 1949 | 29 | 11.12 | 1.00E-06 |
|  | 8 | GO:0010558 | negative regulation of macromolecule bio... | 1838 | 28 | 10.49 | 1.00E-06 |
|  | 9 | GO:0051253 | negative regulation of RNA metabolic pro... | 1415 | 24 | 8.08 | 1.20E-06 |
|  | 10 | GO:2000113 | negative regulation of cellular macromol... | 1760 | 27 | 10.05 | 1.50E-06 |
|  | 11 | GO:0045934 | negative regulation of nucleobase-contai... | 1575 | 25 | 8.99 | 2.20E-06 |
|  | 12 | GO:0010605 | negative regulation of macromolecule met... | 3147 | 38 | 17.96 | 2.50E-06 |
|  | 13 | GO:0031327 | negative regulation of cellular biosynth... | 1929 | 28 | 11.01 | 2.70E-06 |
|  | 14 | GO:0009888 | tissue development | 2175 | 30 | 12.41 | 3.10E-06 |
|  | 15 | GO:0048523 | negative regulation of cellular process | 5330 | 53 | 30.42 | 3.90E-06 |
|  | 16 | GO:0031325 | positive regulation of cellular metaboli... | 3351 | 39 | 19.13 | 4.30E-06 |
|  | 17 | GO:0048522 | positive regulation of cellular process | 5680 | 55 | 32.42 | 5.10E-06 |
|  | 18 | GO:0098532 | histone H3-K27 trimethylation | 7 | 3 | 0.04 | 6.20E-06 |
|  | 19 | GO:0009892 | negative regulation of metabolic process | 3408 | 39 | 19.45 | 6.50E-06 |
|  | 20 | GO:0006357 | regulation of transcription by RNA polym... | 2406 | 31 | 13.73 | 8.20E-06 |
|  | 21 | GO:0048519 | negative regulation of biological proces... | 5929 | 56 | 33.84 | 8.70E-06 |
|  | 22 | GO:0016584 | nucleosome positioning | 8 | 3 | 0.05 | 9.90E-06 |
|  | 23 | GO:0048518 | positive regulation of biological proces... | 6433 | 59 | 36.72 | 1.00E-05 |
|  | 24 | GO:0009893 | positive regulation of metabolic process | 3615 | 40 | 20.63 | 1.10E-05 |
|  | 25 | GO:0012501 | programmed cell death | 2317 | 30 | 13.22 | 1.10E-05 |
|  | 26 | GO:0009605 | response to external stimulus | 2452 | 31 | 14 | 1.20E-05 |
|  | 27 | GO:0008219 | cell death | 2453 | 31 | 14 | 1.20E-05 |
|  | 28 | GO:0051173 | positive regulation of nitrogen compound... | 3234 | 37 | 18.46 | 1.30E-05 |
|  | 29 | GO:0006366 | transcription by RNA polymerase II | 2599 | 32 | 14.83 | 1.40E-05 |
|  | 30 | GO:0045944 | positive regulation of transcription by ... | 1216 | 20 | 6.94 | 1.60E-05 |
|  | 31 | GO:0048856 | anatomical structure development | 6364 | 58 | 36.32 | 1.70E-05 |
|  | 32 | GO:0019731 | antibacterial humoral response | 56 | 5 | 0.32 | 1.70E-05 |
|  | 33 | GO:0002227 | innate immune response in mucosa | 31 | 4 | 0.18 | 2.80E-05 |
|  | 34 | GO:0048627 | myoblast development | 2 | 2 | 0.01 | 3.20E-05 |
|  | 35 | GO:0010604 | positive regulation of macromolecule met... | 3379 | 37 | 19.29 | 3.40E-05 |
|  | 36 | GO:0042127 | regulation of cell proliferation | 1863 | 25 | 10.63 | 4.00E-05 |
|  | 37 | GO:0045935 | positive regulation of nucleobase-contai... | 1870 | 25 | 10.67 | 4.30E-05 |
|  | 38 | GO:0006334 | nucleosome assembly | 164 | 7 | 0.94 | 4.30E-05 |
|  | 39 | GO:0051254 | positive regulation of RNA metabolic pro... | 1638 | 23 | 9.35 | 4.40E-05 |
|  | 40 | GO:0006351 | transcription, DNA-templated | 4287 | 43 | 24.47 | 5.10E-05 |
|  | 41 | GO:0044260 | cellular macromolecule metabolic process | 9319 | 74 | 53.19 | 5.40E-05 |
|  | 42 | GO:0008544 | epidermis development | 536 | 12 | 3.06 | 5.60E-05 |
|  | 43 | GO:0097659 | nucleic acid-templated transcription | 4305 | 43 | 24.57 | 5.70E-05 |
|  | 44 | GO:0045893 | positive regulation of transcription, DN... | 1554 | 22 | 8.87 | 5.80E-05 |
|  | 45 | GO:1903508 | positive regulation of nucleic acid-temp... | 1554 | 22 | 8.87 | 5.80E-05 |
|  | 46 | GO:1902680 | positive regulation of RNA biosynthetic ... | 1555 | 22 | 8.88 | 5.90E-05 |
|  | 47 | GO:0032774 | RNA biosynthetic process | 4327 | 43 | 24.7 | 6.40E-05 |
|  | 48 | GO:0006915 | apoptotic process | 2170 | 27 | 12.39 | 6.90E-05 |
|  | 49 | GO:0008283 | cell proliferation | 2299 | 28 | 13.12 | 7.10E-05 |
|  | 50 | GO:0032502 | developmental process | 6840 | 59 | 39.04 | 7.90E-05 |
| CC | 1 | GO:0000786 | nucleosome | 108 | 8 | 0.58 | 1.20E-07 |
|  | 2 | GO:0044815 | DNA packaging complex | 115 | 8 | 0.61 | 1.90E-07 |
|  | 3 | GO:0005634 | nucleus | 7961 | 68 | 42.57 | 8.20E-07 |
|  | 4 | GO:0043226 | organelle | 15040 | 99 | 80.42 | 1.50E-05 |
|  | 5 | GO:0032993 | protein-DNA complex | 206 | 8 | 1.1 | 1.50E-05 |
|  | 6 | GO:0043227 | membrane-bounded organelle | 13959 | 93 | 74.64 | 6.50E-05 |
|  | 7 | GO:0000785 | chromatin | 596 | 12 | 3.19 | 8.30E-05 |
|  | 8 | GO:0070062 | extracellular exosome | 3105 | 31 | 16.6 | 0.00031 |
|  | 9 | GO:1903561 | extracellular vesicle | 3127 | 31 | 16.72 | 0.00035 |
|  | 10 | GO:0043230 | extracellular organelle | 3129 | 31 | 16.73 | 0.00036 |
|  | 11 | GO:0043231 | intracellular membrane-bounded organelle | 12050 | 82 | 64.44 | 0.00037 |
|  | 12 | GO:0044424 | intracellular part | 15901 | 99 | 85.03 | 0.00057 |
|  | 13 | GO:0043229 | intracellular organelle | 13979 | 90 | 74.75 | 0.00091 |
|  | 14 | GO:0000790 | nuclear chromatin | 408 | 8 | 2.18 | 0.00158 |
|  | 15 | GO:0005622 | intracellular | 16208 | 99 | 86.67 | 0.00183 |
|  | 16 | GO:0043228 | non-membrane-bounded organelle | 4552 | 38 | 24.34 | 0.00192 |
|  | 17 | GO:0043232 | intracellular non-membrane-bounded organ... | 4552 | 38 | 24.34 | 0.00192 |
|  | 18 | GO:0044427 | chromosomal part | 991 | 13 | 5.3 | 0.00244 |
|  | 19 | GO:0005615 | extracellular space | 4339 | 36 | 23.2 | 0.00298 |
|  | 20 | GO:0005667 | transcription factor complex | 373 | 7 | 1.99 | 0.00393 |
|  | 21 | GO:0005912 | adherens junction | 604 | 9 | 3.23 | 0.00513 |
|  | 22 | GO:0098745 | Dcp1-Dcp2 complex | 1 | 1 | 0.01 | 0.00535 |
|  | 23 | GO:0005829 | cytosol | 5508 | 42 | 29.45 | 0.00587 |
|  | 24 | GO:0070161 | anchoring junction | 627 | 9 | 3.35 | 0.00651 |
|  | 25 | GO:0005719 | nuclear euchromatin | 24 | 2 | 0.13 | 0.00724 |
|  | 26 | GO:0044421 | extracellular region part | 4578 | 36 | 24.48 | 0.0074 |
|  | 27 | GO:0005694 | chromosome | 1132 | 13 | 6.05 | 0.00742 |
|  | 28 | GO:0097057 | TRAF2-GSTP1 complex | 2 | 1 | 0.01 | 0.01067 |
|  | 29 | GO:0031981 | nuclear lumen | 4533 | 35 | 24.24 | 0.0112 |
|  | 30 | GO:0000791 | euchromatin | 33 | 2 | 0.18 | 0.01343 |
|  | 31 | GO:0020016 | ciliary pocket | 3 | 1 | 0.02 | 0.01596 |
|  | 32 | GO:0020018 | ciliary pocket membrane | 3 | 1 | 0.02 | 0.01596 |
|  | 33 | GO:0097013 | phagocytic vesicle lumen | 3 | 1 | 0.02 | 0.01596 |
|  | 34 | GO:1905286 | serine-type peptidase complex | 3 | 1 | 0.02 | 0.01596 |
|  | 35 | GO:0044454 | nuclear chromosome part | 628 | 8 | 3.36 | 0.01952 |
|  | 36 | GO:0044464 | cell part | 18844 | 107 | 100.77 | 0.02028 |
|  | 37 | GO:0035517 | PR-DUB complex | 4 | 1 | 0.02 | 0.02122 |
|  | 38 | GO:0072558 | NLRP1 inflammasome complex | 4 | 1 | 0.02 | 0.02122 |
|  | 39 | GO:0000788 | nuclear nucleosome | 42 | 2 | 0.22 | 0.02123 |
|  | 40 | GO:0044428 | nuclear part | 4922 | 36 | 26.32 | 0.02263 |
|  | 41 | GO:0031974 | membrane-enclosed lumen | 5776 | 41 | 30.89 | 0.02272 |
|  | 42 | GO:0043233 | organelle lumen | 5776 | 41 | 30.89 | 0.02272 |
|  | 43 | GO:0070013 | intracellular organelle lumen | 5776 | 41 | 30.89 | 0.02272 |
|  | 44 | GO:0005623 | cell | 18876 | 107 | 100.94 | 0.02292 |
|  | 45 | GO:0005576 | extracellular region | 5443 | 39 | 29.11 | 0.02339 |
|  | 46 | GO:0099512 | supramolecular fiber | 1046 | 11 | 5.59 | 0.02427 |
|  | 47 | GO:0044798 | nuclear transcription factor complex | 205 | 4 | 1.1 | 0.0244 |
|  | 48 | GO:0010494 | cytoplasmic stress granule | 46 | 2 | 0.25 | 0.02517 |
|  | 49 | GO:0099081 | supramolecular polymer | 1053 | 11 | 5.63 | 0.02533 |
|  | 50 | GO:0099080 | supramolecular complex | 1054 | 11 | 5.64 | 0.02548 |
| MF | 1 | GO:0003677 | DNA binding | 2693 | 37 | 15.24 | 1.10E-07 |
|  | 2 | GO:0000982 | transcription factor activity, RNA polym... | 424 | 14 | 2.4 | 1.20E-07 |
|  | 3 | GO:0044212 | transcription regulatory region DNA bind... | 943 | 20 | 5.34 | 2.90E-07 |
|  | 4 | GO:0001067 | regulatory region nucleic acid binding | 944 | 20 | 5.34 | 3.00E-07 |
|  | 5 | GO:0000977 | RNA polymerase II regulatory region sequ... | 697 | 17 | 3.94 | 3.80E-07 |
|  | 6 | GO:0000976 | transcription regulatory region sequence... | 783 | 18 | 4.43 | 3.90E-07 |
|  | 7 | GO:0001012 | RNA polymerase II regulatory region DNA ... | 700 | 17 | 3.96 | 4.00E-07 |
|  | 8 | GO:0043565 | sequence-specific DNA binding | 1195 | 22 | 6.76 | 7.70E-07 |
|  | 9 | GO:1990837 | sequence-specific double-stranded DNA bi... | 821 | 18 | 4.64 | 7.80E-07 |
|  | 10 | GO:0003690 | double-stranded DNA binding | 920 | 19 | 5.2 | 9.10E-07 |
|  | 11 | GO:0000981 | RNA polymerase II transcription factor a... | 1163 | 20 | 6.58 | 7.40E-06 |
|  | 12 | GO:0005515 | protein binding | 13002 | 94 | 73.56 | 7.80E-06 |
|  | 13 | GO:0000978 | RNA polymerase II proximal promoter sequ... | 447 | 12 | 2.53 | 8.60E-06 |
|  | 14 | GO:0000987 | proximal promoter sequence-specific DNA ... | 463 | 12 | 2.62 | 1.20E-05 |
|  | 15 | GO:0001077 | transcriptional activator activity, RNA ... | 283 | 9 | 1.6 | 3.30E-05 |
|  | 16 | GO:0001228 | transcriptional activator activity, RNA ... | 440 | 11 | 2.49 | 4.00E-05 |
|  | 17 | GO:0031490 | chromatin DNA binding | 114 | 6 | 0.64 | 4.60E-05 |
|  | 18 | GO:0008134 | transcription factor binding | 661 | 13 | 3.74 | 9.20E-05 |
|  | 19 | GO:0001227 | transcriptional repressor activity, RNA ... | 249 | 7 | 1.41 | 0.00054 |
|  | 20 | GO:0003682 | chromatin binding | 601 | 11 | 3.4 | 0.0006 |
|  | 21 | GO:1901363 | heterocyclic compound binding | 6641 | 54 | 37.57 | 0.00084 |
|  | 22 | GO:0003700 | DNA binding transcription factor activit... | 1646 | 20 | 9.31 | 0.00086 |
|  | 23 | GO:0046982 | protein heterodimerization activity | 531 | 10 | 3 | 0.00086 |
|  | 24 | GO:0003676 | nucleic acid binding | 4589 | 41 | 25.96 | 0.00088 |
|  | 25 | GO:0140110 | transcription regulator activity | 2041 | 23 | 11.55 | 0.00094 |
|  | 26 | GO:0097159 | organic cyclic compound binding | 6732 | 54 | 38.09 | 0.00121 |
|  | 27 | GO:0042910 | xenobiotic transmembrane transporter act... | 10 | 2 | 0.06 | 0.00139 |
|  | 28 | GO:0001078 | transcriptional repressor activity, RNA ... | 145 | 5 | 0.82 | 0.00141 |
|  | 29 | GO:0003810 | protein-glutamine gamma-glutamyltransfer... | 11 | 2 | 0.06 | 0.00169 |
|  | 30 | GO:0001046 | core promoter sequence-specific DNA bind... | 90 | 4 | 0.51 | 0.00171 |
|  | 31 | GO:0005488 | binding | 16612 | 104 | 93.98 | 0.00227 |
|  | 32 | GO:0001222 | transcription corepressor binding | 14 | 2 | 0.08 | 0.00276 |
|  | 33 | GO:0005523 | tropomyosin binding | 14 | 2 | 0.08 | 0.00276 |
|  | 34 | GO:0042043 | neurexin family protein binding | 14 | 2 | 0.08 | 0.00276 |
|  | 35 | GO:0097153 | cysteine-type endopeptidase activity inv... | 16 | 2 | 0.09 | 0.00361 |
|  | 36 | GO:0046983 | protein dimerization activity | 1386 | 16 | 7.84 | 0.00489 |
|  | 37 | GO:0016755 | transferase activity, transferring amino... | 19 | 2 | 0.11 | 0.00509 |
|  | 38 | GO:0004855 | xanthine oxidase activity | 1 | 1 | 0.01 | 0.00566 |
|  | 39 | GO:0005368 | taurine transmembrane transporter activi... | 1 | 1 | 0.01 | 0.00566 |
|  | 40 | GO:0005369 | taurine:sodium symporter activity | 1 | 1 | 0.01 | 0.00566 |
|  | 41 | GO:0016727 | oxidoreductase activity, acting on CH or... | 1 | 1 | 0.01 | 0.00566 |
|  | 42 | GO:0017024 | myosin I binding | 1 | 1 | 0.01 | 0.00566 |
|  | 43 | GO:0031700 | adrenomedullin receptor binding | 1 | 1 | 0.01 | 0.00566 |
|  | 44 | GO:0035538 | carbohydrate response element binding | 1 | 1 | 0.01 | 0.00566 |
|  | 45 | GO:0035730 | S-nitrosoglutathione binding | 1 | 1 | 0.01 | 0.00566 |
|  | 46 | GO:0035731 | dinitrosyl-iron complex binding | 1 | 1 | 0.01 | 0.00566 |
|  | 47 | GO:0036403 | arachidonate 8(S)-lipoxygenase activity | 1 | 1 | 0.01 | 0.00566 |
|  | 48 | GO:0071820 | N-box binding | 1 | 1 | 0.01 | 0.00566 |
|  | 49 | GO:0001047 | core promoter binding | 150 | 4 | 0.85 | 0.01045 |
|  | 50 | GO:0071889 | 14-3-3 protein binding | 28 | 2 | 0.16 | 0.01089 |
