# Supplementary material for: Transcriptional profiling to identify the key genes and pathways of pterygium
Source: PeerJ. 2020 May 4;8:e9056. doi: 10.7717/peerj.9056 (PMC7204871; doi:10.7717/peerj.9056)
Supplement: Table S3 [file peerj-08-9056-s003.docx]

**Supplementary table3 Gene Ontology of each module in the WGCNA analysis**

| **Module** | **Function** | **Rank** | **GO.ID** | **Term** | **Annotated** | **Significant** | **Expected** | **Classic Fisher** |
| --- | --- | --- | --- | --- | --- | --- | --- | --- |
| Blue | BP | 1 | GO:0043062 | extracellular structure organization | 445 | 17 | 1.7 | 7.30E-13 |
|  |  | 2 | GO:0030198 | extracellular matrix organization | 384 | 16 | 1.47 | 1.00E-12 |
|  |  | 3 | GO:0016477 | cell migration | 1537 | 24 | 5.87 | 1.20E-09 |
|  |  | 4 | GO:0048870 | cell motility | 1684 | 24 | 6.44 | 7.60E-09 |
|  |  | 5 | GO:0051674 | localization of cell | 1684 | 24 | 6.44 | 7.60E-09 |
|  |  | 6 | GO:0040011 | locomotion | 1955 | 25 | 7.47 | 2.90E-08 |
|  |  | 7 | GO:0006928 | movement of cell or subcellular componen... | 2185 | 26 | 8.35 | 6.10E-08 |
|  |  | 8 | GO:0051270 | regulation of cellular component movemen... | 1056 | 18 | 4.04 | 6.40E-08 |
|  |  | 9 | GO:0051239 | regulation of multicellular organismal p... | 3303 | 32 | 12.62 | 1.20E-07 |
|  |  | 10 | GO:0001568 | blood vessel development | 765 | 15 | 2.92 | 1.60E-07 |
|  |  | 11 | GO:0032502 | developmental process | 6840 | 48 | 26.14 | 2.50E-07 |
|  |  | 12 | GO:0001944 | vasculature development | 790 | 15 | 3.02 | 2.50E-07 |
|  |  | 13 | GO:0030334 | regulation of cell migration | 917 | 16 | 3.5 | 2.90E-07 |
|  |  | 14 | GO:0072358 | cardiovascular system development | 801 | 15 | 3.06 | 3.00E-07 |
|  |  | 15 | GO:0071559 | response to transforming growth factor b... | 246 | 9 | 0.94 | 4.00E-07 |
|  |  | 16 | GO:2000145 | regulation of cell motility | 970 | 16 | 3.71 | 6.10E-07 |
|  |  | 17 | GO:0050793 | regulation of developmental process | 2807 | 28 | 10.73 | 6.40E-07 |
|  |  | 18 | GO:0001101 | response to acid chemical | 356 | 10 | 1.36 | 9.90E-07 |
|  |  | 19 | GO:0072359 | circulatory system development | 1155 | 17 | 4.41 | 1.30E-06 |
|  |  | 20 | GO:0022610 | biological adhesion | 1588 | 20 | 6.07 | 1.40E-06 |
|  |  | 21 | GO:0030155 | regulation of cell adhesion | 809 | 14 | 3.09 | 2.00E-06 |
|  |  | 22 | GO:0048731 | system development | 5191 | 39 | 19.84 | 2.10E-06 |
|  |  | 23 | GO:0040012 | regulation of locomotion | 1076 | 16 | 4.11 | 2.40E-06 |
|  |  | 24 | GO:0070848 | response to growth factor | 731 | 13 | 2.79 | 3.60E-06 |
|  |  | 25 | GO:0071560 | cellular response to transforming growth... | 242 | 8 | 0.92 | 3.90E-06 |
|  |  | 26 | GO:0009653 | anatomical structure morphogenesis | 2903 | 27 | 11.1 | 4.40E-06 |
|  |  | 27 | GO:0007155 | cell adhesion | 1580 | 19 | 6.04 | 5.30E-06 |
|  |  | 28 | GO:0007275 | multicellular organism development | 5844 | 41 | 22.34 | 5.90E-06 |
|  |  | 29 | GO:0033483 | gas homeostasis | 10 | 3 | 0.04 | 6.30E-06 |
|  |  | 30 | GO:0031589 | cell-substrate adhesion | 350 | 9 | 1.34 | 7.30E-06 |
|  |  | 31 | GO:0048856 | anatomical structure development | 6364 | 43 | 24.32 | 7.60E-06 |
|  |  | 32 | GO:0007166 | cell surface receptor signaling pathway | 3382 | 29 | 12.93 | 8.50E-06 |
|  |  | 33 | GO:0023052 | signaling | 7642 | 48 | 29.21 | 9.70E-06 |
|  |  | 34 | GO:0007154 | cell communication | 7659 | 48 | 29.27 | 1.00E-05 |
|  |  | 35 | GO:0001570 | vasculogenesis | 78 | 5 | 0.3 | 1.20E-05 |
|  |  | 36 | GO:0071363 | cellular response to growth factor stimu... | 705 | 12 | 2.69 | 1.40E-05 |
|  |  | 37 | GO:0032501 | multicellular organismal process | 8327 | 50 | 31.83 | 1.90E-05 |
|  |  | 38 | GO:0071229 | cellular response to acid chemical | 218 | 7 | 0.83 | 2.00E-05 |
|  |  | 39 | GO:0032879 | regulation of localization | 2964 | 26 | 11.33 | 2.10E-05 |
|  |  | 40 | GO:0007165 | signal transduction | 7095 | 45 | 27.12 | 2.20E-05 |
|  |  | 41 | GO:0048545 | response to steroid hormone | 425 | 9 | 1.62 | 3.40E-05 |
|  |  | 42 | GO:0033484 | nitric oxide homeostasis | 3 | 2 | 0.01 | 4.30E-05 |
|  |  | 43 | GO:2000026 | regulation of multicellular organismal d... | 2173 | 21 | 8.31 | 4.30E-05 |
|  |  | 44 | GO:0048869 | cellular developmental process | 4750 | 34 | 18.15 | 5.00E-05 |
|  |  | 45 | GO:0048514 | blood vessel morphogenesis | 682 | 11 | 2.61 | 5.30E-05 |
|  |  | 46 | GO:0031960 | response to corticosteroid | 176 | 6 | 0.67 | 5.80E-05 |
|  |  | 47 | GO:0009719 | response to endogenous stimulus | 1720 | 18 | 6.57 | 6.40E-05 |
|  |  | 48 | GO:0007167 | enzyme linked receptor protein signaling... | 1107 | 14 | 4.23 | 6.70E-05 |
|  |  | 49 | GO:0048513 | animal organ development | 3789 | 29 | 14.48 | 7.70E-05 |
|  |  | 50 | GO:0043200 | response to amino acid | 121 | 5 | 0.46 | 1.00E-04 |
|  | CC | 1 | GO:0044420 | extracellular matrix component | 132 | 11 | 0.49 | 2.10E-12 |
|  |  | 2 | GO:0005578 | proteinaceous extracellular matrix | 410 | 15 | 1.52 | 2.40E-11 |
|  |  | 3 | GO:0031012 | extracellular matrix | 519 | 16 | 1.93 | 6.20E-11 |
|  |  | 4 | GO:0005576 | extracellular region | 5443 | 42 | 20.19 | 1.20E-07 |
|  |  | 5 | GO:0044421 | extracellular region part | 4578 | 37 | 16.98 | 4.20E-07 |
|  |  | 6 | GO:0005615 | extracellular space | 4339 | 35 | 16.1 | 1.20E-06 |
|  |  | 7 | GO:0031982 | vesicle | 4920 | 37 | 18.25 | 2.70E-06 |
|  |  | 8 | GO:0005584 | collagen type I trimer | 2 | 2 | 0.01 | 1.40E-05 |
|  |  | 9 | GO:0005604 | basement membrane | 92 | 5 | 0.34 | 2.40E-05 |
|  |  | 10 | GO:0005583 | fibrillar collagen trimer | 25 | 3 | 0.09 | 0.00011 |
|  |  | 11 | GO:0098643 | banded collagen fibril | 25 | 3 | 0.09 | 0.00011 |
|  |  | 12 | GO:0009986 | cell surface | 936 | 12 | 3.47 | 0.00016 |
|  |  | 13 | GO:0070062 | extracellular exosome | 3105 | 24 | 11.52 | 0.00024 |
|  |  | 14 | GO:0098644 | complex of collagen trimers | 33 | 3 | 0.12 | 0.00025 |
|  |  | 15 | GO:1903561 | extracellular vesicle | 3127 | 24 | 11.6 | 0.00027 |
|  |  | 16 | GO:0043230 | extracellular organelle | 3129 | 24 | 11.61 | 0.00028 |
|  |  | 17 | GO:0031410 | cytoplasmic vesicle | 2636 | 21 | 9.78 | 0.00046 |
|  |  | 18 | GO:0044459 | plasma membrane part | 3442 | 25 | 12.77 | 0.00046 |
|  |  | 19 | GO:0097708 | intracellular vesicle | 2639 | 21 | 9.79 | 0.00046 |
|  |  | 20 | GO:0005581 | collagen trimer | 101 | 4 | 0.37 | 0.00054 |
|  |  | 21 | GO:0005887 | integral component of plasma membrane | 2106 | 18 | 7.81 | 0.00057 |
|  |  | 22 | GO:0001527 | microfibril | 10 | 2 | 0.04 | 0.0006 |
|  |  | 23 | GO:0099503 | secretory vesicle | 1106 | 12 | 4.1 | 0.00073 |
|  |  | 24 | GO:0030141 | secretory granule | 959 | 11 | 3.56 | 0.00079 |
|  |  | 25 | GO:0009897 | external side of plasma membrane | 295 | 6 | 1.09 | 0.0008 |
|  |  | 26 | GO:0031226 | intrinsic component of plasma membrane | 2174 | 18 | 8.06 | 0.00083 |
|  |  | 27 | GO:0005788 | endoplasmic reticulum lumen | 317 | 6 | 1.18 | 0.00116 |
|  |  | 28 | GO:0071944 | cell periphery | 6532 | 37 | 24.23 | 0.0017 |
|  |  | 29 | GO:0005886 | plasma membrane | 6420 | 36 | 23.81 | 0.00254 |
|  |  | 30 | GO:0031093 | platelet alpha granule lumen | 74 | 3 | 0.27 | 0.00263 |
|  |  | 31 | GO:0005605 | basal lamina | 21 | 2 | 0.08 | 0.00272 |
|  |  | 32 | GO:0005586 | collagen type III trimer | 1 | 1 | 0 | 0.00371 |
|  |  | 33 | GO:0005596 | collagen type XIV trimer | 1 | 1 | 0 | 0.00371 |
|  |  | 34 | GO:0036053 | glomerular endothelium fenestra | 1 | 1 | 0 | 0.00371 |
|  |  | 35 | GO:0045177 | apical part of cell | 404 | 6 | 1.5 | 0.00388 |
|  |  | 36 | GO:0099512 | supramolecular fiber | 1046 | 10 | 3.88 | 0.00517 |
|  |  | 37 | GO:0099081 | supramolecular polymer | 1053 | 10 | 3.91 | 0.00541 |
|  |  | 38 | GO:0099080 | supramolecular complex | 1054 | 10 | 3.91 | 0.00545 |
|  |  | 39 | GO:0031091 | platelet alpha granule | 103 | 3 | 0.38 | 0.00665 |
|  |  | 40 | GO:0005775 | vacuolar lumen | 203 | 4 | 0.75 | 0.00689 |
|  |  | 41 | GO:0030027 | lamellipodium | 204 | 4 | 0.76 | 0.00701 |
|  |  | 42 | GO:0034681 | integrin alpha11-beta1 complex | 2 | 1 | 0.01 | 0.00741 |
|  |  | 43 | GO:0097057 | TRAF2-GSTP1 complex | 2 | 1 | 0.01 | 0.00741 |
|  |  | 44 | GO:1990682 | CSF1-CSF1R complex | 2 | 1 | 0.01 | 0.00741 |
|  |  | 45 | GO:0043202 | lysosomal lumen | 112 | 3 | 0.42 | 0.00837 |
|  |  | 46 | GO:0005911 | cell-cell junction | 479 | 6 | 1.78 | 0.00873 |
|  |  | 47 | GO:0030667 | secretory granule membrane | 357 | 5 | 1.32 | 0.01053 |
|  |  | 48 | GO:0030934 | anchoring collagen complex | 3 | 1 | 0.01 | 0.01109 |
|  |  | 49 | GO:0030315 | T-tubule | 46 | 2 | 0.17 | 0.01265 |
|  |  | 50 | GO:0034774 | secretory granule lumen | 374 | 5 | 1.39 | 0.01268 |
|  | MF | 1 | GO:0019838 | growth factor binding | 145 | 7 | 0.51 | 8.10E-07 |
|  |  | 2 | GO:0005201 | extracellular matrix structural constitu... | 95 | 6 | 0.34 | 1.10E-06 |
|  |  | 3 | GO:1901681 | sulfur compound binding | 262 | 8 | 0.93 | 4.00E-06 |
|  |  | 4 | GO:0048407 | platelet-derived growth factor binding | 11 | 3 | 0.04 | 6.90E-06 |
|  |  | 5 | GO:0005518 | collagen binding | 79 | 5 | 0.28 | 8.90E-06 |
|  |  | 6 | GO:0019955 | cytokine binding | 116 | 5 | 0.41 | 5.70E-05 |
|  |  | 7 | GO:0002020 | protease binding | 169 | 5 | 0.6 | 0.00034 |
|  |  | 8 | GO:0005539 | glycosaminoglycan binding | 240 | 5 | 0.85 | 0.00162 |
|  |  | 9 | GO:0050431 | transforming growth factor beta binding | 20 | 2 | 0.07 | 0.00226 |
|  |  | 10 | GO:0042813 | Wnt-activated receptor activity | 23 | 2 | 0.08 | 0.00299 |
|  |  | 11 | GO:0043178 | alcohol binding | 81 | 3 | 0.29 | 0.00299 |
|  |  | 12 | GO:0019901 | protein kinase binding | 708 | 8 | 2.51 | 0.00351 |
|  |  | 13 | GO:0035730 | S-nitrosoglutathione binding | 1 | 1 | 0 | 0.00355 |
|  |  | 14 | GO:0035731 | dinitrosyl-iron complex binding | 1 | 1 | 0 | 0.00355 |
|  |  | 15 | GO:0070320 | inward rectifier potassium channel inhib... | 1 | 1 | 0 | 0.00355 |
|  |  | 16 | GO:0001968 | fibronectin binding | 26 | 2 | 0.09 | 0.00382 |
|  |  | 17 | GO:0008201 | heparin binding | 184 | 4 | 0.65 | 0.00416 |
|  |  | 18 | GO:0030674 | protein binding, bridging | 185 | 4 | 0.66 | 0.00424 |
|  |  | 19 | GO:0005509 | calcium ion binding | 752 | 8 | 2.67 | 0.00503 |
|  |  | 20 | GO:0017147 | Wnt-protein binding | 32 | 2 | 0.11 | 0.00575 |
|  |  | 21 | GO:0060090 | molecular adaptor activity | 206 | 4 | 0.73 | 0.00619 |
|  |  | 22 | GO:0019900 | kinase binding | 788 | 8 | 2.8 | 0.00663 |
|  |  | 23 | GO:0003870 | 5-aminolevulinate synthase activity | 2 | 1 | 0.01 | 0.00709 |
|  |  | 24 | GO:0004958 | prostaglandin F receptor activity | 2 | 1 | 0.01 | 0.00709 |
|  |  | 25 | GO:0005011 | macrophage colony-stimulating factor rec... | 2 | 1 | 0.01 | 0.00709 |
|  |  | 26 | GO:0008147 | structural constituent of bone | 2 | 1 | 0.01 | 0.00709 |
|  |  | 27 | GO:0016749 | N-succinyltransferase activity | 2 | 1 | 0.01 | 0.00709 |
|  |  | 28 | GO:0030984 | kininogen binding | 2 | 1 | 0.01 | 0.00709 |
|  |  | 29 | GO:0050135 | NAD(P)+ nucleosidase activity | 2 | 1 | 0.01 | 0.00709 |
|  |  | 30 | GO:0050436 | microfibril binding | 2 | 1 | 0.01 | 0.00709 |
|  |  | 31 | GO:0061809 | NAD+ nucleotidase, cyclic ADP-ribose gen... | 2 | 1 | 0.01 | 0.00709 |
|  |  | 32 | GO:0070026 | nitric oxide binding | 2 | 1 | 0.01 | 0.00709 |
|  |  | 33 | GO:0097718 | disordered domain specific binding | 37 | 2 | 0.13 | 0.00763 |
|  |  | 34 | GO:0005198 | structural molecule activity | 809 | 8 | 2.87 | 0.00772 |
|  |  | 35 | GO:0016504 | peptidase activator activity | 39 | 2 | 0.14 | 0.00845 |
|  |  | 36 | GO:0043394 | proteoglycan binding | 39 | 2 | 0.14 | 0.00845 |
|  |  | 37 | GO:0044325 | ion channel binding | 123 | 3 | 0.44 | 0.00956 |
|  |  | 38 | GO:0003953 | NAD+ nucleosidase activity | 3 | 1 | 0.01 | 0.01061 |
|  |  | 39 | GO:0004992 | platelet activating factor receptor acti... | 3 | 1 | 0.01 | 0.01061 |
|  |  | 40 | GO:0008597 | calcium-dependent protein serine/threoni... | 3 | 1 | 0.01 | 0.01061 |
|  |  | 41 | GO:0016748 | succinyltransferase activity | 3 | 1 | 0.01 | 0.01061 |
|  |  | 42 | GO:0019959 | interleukin-8 binding | 3 | 1 | 0.01 | 0.01061 |
|  |  | 43 | GO:0031997 | N-terminal myristoylation domain binding | 3 | 1 | 0.01 | 0.01061 |
|  |  | 44 | GO:0015485 | cholesterol binding | 44 | 2 | 0.16 | 0.01067 |
|  |  | 45 | GO:0061134 | peptidase regulator activity | 247 | 4 | 0.88 | 0.01153 |
|  |  | 46 | GO:0004177 | aminopeptidase activity | 46 | 2 | 0.16 | 0.01162 |
|  |  | 47 | GO:0032934 | sterol binding | 49 | 2 | 0.17 | 0.01311 |
|  |  | 48 | GO:0000298 | endopolyphosphatase activity | 4 | 1 | 0.01 | 0.01412 |
|  |  | 49 | GO:0008486 | diphosphoinositol-polyphosphate diphosph... | 4 | 1 | 0.01 | 0.01412 |
|  |  | 50 | GO:0016807 | cysteine-type carboxypeptidase activity | 4 | 1 | 0.01 | 0.01412 |
| Brown | BP | 1 | GO:0016043 | cellular component organization | 6846 | 27 | 15 | 0.00017 |
|  |  | 2 | GO:0071840 | cellular component organization or bioge... | 7101 | 27 | 15.56 | 0.00035 |
|  |  | 3 | GO:0033622 | integrin activation | 19 | 2 | 0.04 | 0.00078 |
|  |  | 4 | GO:0090280 | positive regulation of calcium ion impor... | 19 | 2 | 0.04 | 0.00078 |
|  |  | 5 | GO:2001025 | positive regulation of response to drug | 28 | 2 | 0.06 | 0.00171 |
|  |  | 6 | GO:0070367 | negative regulation of hepatocyte differ... | 1 | 1 | 0 | 0.00219 |
|  |  | 7 | GO:1990478 | response to ultrasound | 1 | 1 | 0 | 0.00219 |
|  |  | 8 | GO:0072659 | protein localization to plasma membrane | 258 | 4 | 0.57 | 0.00241 |
|  |  | 9 | GO:0048846 | axon extension involved in axon guidance | 35 | 2 | 0.08 | 0.00267 |
|  |  | 10 | GO:1902284 | neuron projection extension involved in ... | 35 | 2 | 0.08 | 0.00267 |
|  |  | 11 | GO:1902742 | apoptotic process involved in developmen... | 35 | 2 | 0.08 | 0.00267 |
|  |  | 12 | GO:0009968 | negative regulation of signal transducti... | 1418 | 9 | 3.11 | 0.00314 |
|  |  | 13 | GO:1990778 | protein localization to cell periphery | 290 | 4 | 0.64 | 0.00366 |
|  |  | 14 | GO:0090279 | regulation of calcium ion import | 42 | 2 | 0.09 | 0.00382 |
|  |  | 15 | GO:0098815 | modulation of excitatory postsynaptic po... | 43 | 2 | 0.09 | 0.004 |
|  |  | 16 | GO:0097435 | supramolecular fiber organization | 707 | 6 | 1.55 | 0.00419 |
|  |  | 17 | GO:0070366 | regulation of hepatocyte differentiation | 2 | 1 | 0 | 0.00438 |
|  |  | 18 | GO:0090038 | negative regulation of protein kinase C ... | 2 | 1 | 0 | 0.00438 |
|  |  | 19 | GO:1903756 | regulation of transcription from RNA pol... | 2 | 1 | 0 | 0.00438 |
|  |  | 20 | GO:1903758 | negative regulation of transcription fro... | 2 | 1 | 0 | 0.00438 |
|  |  | 21 | GO:1905665 | positive regulation of calcium ion impor... | 2 | 1 | 0 | 0.00438 |
|  |  | 22 | GO:0090102 | cochlea development | 46 | 2 | 0.1 | 0.00457 |
|  |  | 23 | GO:0061028 | establishment of endothelial barrier | 49 | 2 | 0.11 | 0.00517 |
|  |  | 24 | GO:0010648 | negative regulation of cell communicatio... | 1533 | 9 | 3.36 | 0.00526 |
|  |  | 25 | GO:0023057 | negative regulation of signaling | 1537 | 9 | 3.37 | 0.00535 |
|  |  | 26 | GO:0000904 | cell morphogenesis involved in different... | 751 | 6 | 1.65 | 0.00561 |
|  |  | 27 | GO:0016049 | cell growth | 534 | 5 | 1.17 | 0.00601 |
|  |  | 28 | GO:0046426 | negative regulation of JAK-STAT cascade | 54 | 2 | 0.12 | 0.00624 |
|  |  | 29 | GO:1904893 | negative regulation of STAT cascade | 54 | 2 | 0.12 | 0.00624 |
|  |  | 30 | GO:0001560 | regulation of cell growth by extracellul... | 3 | 1 | 0.01 | 0.00656 |
|  |  | 31 | GO:0003192 | mitral valve formation | 3 | 1 | 0.01 | 0.00656 |
|  |  | 32 | GO:0061364 | apoptotic process involved in luteolysis | 3 | 1 | 0.01 | 0.00656 |
|  |  | 33 | GO:1902897 | regulation of postsynaptic density prote... | 3 | 1 | 0.01 | 0.00656 |
|  |  | 34 | GO:0048468 | cell development | 2201 | 11 | 4.82 | 0.0066 |
|  |  | 35 | GO:0009653 | anatomical structure morphogenesis | 2903 | 13 | 6.36 | 0.00749 |
|  |  | 36 | GO:0006027 | glycosaminoglycan catabolic process | 62 | 2 | 0.14 | 0.00816 |
|  |  | 37 | GO:0046847 | filopodium assembly | 63 | 2 | 0.14 | 0.00842 |
|  |  | 38 | GO:0003273 | cell migration involved in endocardial c... | 4 | 1 | 0.01 | 0.00874 |
|  |  | 39 | GO:1902512 | positive regulation of apoptotic DNA fra... | 4 | 1 | 0.01 | 0.00874 |
|  |  | 40 | GO:1902952 | positive regulation of dendritic spine m... | 4 | 1 | 0.01 | 0.00874 |
|  |  | 41 | GO:1903237 | negative regulation of leukocyte tetheri... | 4 | 1 | 0.01 | 0.00874 |
|  |  | 42 | GO:1905664 | regulation of calcium ion import across ... | 4 | 1 | 0.01 | 0.00874 |
|  |  | 43 | GO:1990410 | adrenomedullin receptor signaling pathwa... | 4 | 1 | 0.01 | 0.00874 |
|  |  | 44 | GO:2000297 | negative regulation of synapse maturatio... | 4 | 1 | 0.01 | 0.00874 |
|  |  | 45 | GO:0030198 | extracellular matrix organization | 384 | 4 | 0.84 | 0.00977 |
|  |  | 46 | GO:0006026 | aminoglycan catabolic process | 69 | 2 | 0.15 | 0.01003 |
|  |  | 47 | GO:0001885 | endothelial cell development | 71 | 2 | 0.16 | 0.01059 |
|  |  | 48 | GO:0007166 | cell surface receptor signaling pathway | 3382 | 14 | 7.41 | 0.01065 |
|  |  | 49 | GO:0001554 | luteolysis | 5 | 1 | 0.01 | 0.01091 |
|  |  | 50 | GO:0010991 | negative regulation of SMAD protein comp... | 5 | 1 | 0.01 | 0.01091 |
|  | CC | 1 | GO:0005578 | proteinaceous extracellular matrix | 410 | 8 | 0.87 | 2.10E-06 |
|  |  | 2 | GO:0031012 | extracellular matrix | 519 | 8 | 1.1 | 1.20E-05 |
|  |  | 3 | GO:0097427 | microtubule bundle | 12 | 2 | 0.03 | 0.00029 |
|  |  | 4 | GO:0097630 | intrinsic component of omegasome membran... | 1 | 1 | 0 | 0.00212 |
|  |  | 5 | GO:0097631 | integral component of omegasome membrane | 1 | 1 | 0 | 0.00212 |
|  |  | 6 | GO:0034066 | RIC1-RGP1 guanyl-nucleotide exchange fac... | 2 | 1 | 0 | 0.00423 |
|  |  | 7 | GO:1903143 | adrenomedullin receptor complex | 3 | 1 | 0.01 | 0.00635 |
|  |  | 8 | GO:0071953 | elastic fiber | 4 | 1 | 0.01 | 0.00845 |
|  |  | 9 | GO:1903440 | amylin receptor complex | 4 | 1 | 0.01 | 0.00845 |
|  |  | 10 | GO:0033093 | Weibel-Palade body | 5 | 1 | 0.01 | 0.01055 |
|  |  | 11 | GO:1903439 | calcitonin family receptor complex | 5 | 1 | 0.01 | 0.01055 |
|  |  | 12 | GO:0031093 | platelet alpha granule lumen | 74 | 2 | 0.16 | 0.01077 |
|  |  | 13 | GO:0000127 | transcription factor TFIIIC complex | 6 | 1 | 0.01 | 0.01265 |
|  |  | 14 | GO:1903349 | omegasome membrane | 6 | 1 | 0.01 | 0.01265 |
|  |  | 15 | GO:0005874 | microtubule | 443 | 4 | 0.94 | 0.01417 |
|  |  | 16 | GO:1990462 | omegasome | 7 | 1 | 0.01 | 0.01475 |
|  |  | 17 | GO:0031095 | platelet dense tubular network membrane | 9 | 1 | 0.02 | 0.01892 |
|  |  | 18 | GO:0031091 | platelet alpha granule | 103 | 2 | 0.22 | 0.02014 |
|  |  | 19 | GO:0090576 | RNA polymerase III transcription factor ... | 10 | 1 | 0.02 | 0.021 |
|  |  | 20 | GO:0031094 | platelet dense tubular network | 11 | 1 | 0.02 | 0.02308 |
|  |  | 21 | GO:0072546 | ER membrane protein complex | 11 | 1 | 0.02 | 0.02308 |
|  |  | 22 | GO:0043202 | lysosomal lumen | 112 | 2 | 0.24 | 0.02355 |
|  |  | 23 | GO:0005576 | extracellular region | 5443 | 18 | 11.54 | 0.02382 |
|  |  | 24 | GO:0032045 | guanyl-nucleotide exchange factor comple... | 14 | 1 | 0.03 | 0.02928 |
|  |  | 25 | GO:0038037 | G-protein coupled receptor dimeric compl... | 14 | 1 | 0.03 | 0.02928 |
|  |  | 26 | GO:0097648 | G-protein coupled receptor complex | 14 | 1 | 0.03 | 0.02928 |
|  |  | 27 | GO:0044420 | extracellular matrix component | 132 | 2 | 0.28 | 0.0319 |
|  |  | 28 | GO:0005796 | Golgi lumen | 139 | 2 | 0.29 | 0.03506 |
|  |  | 29 | GO:0005875 | microtubule associated complex | 160 | 2 | 0.34 | 0.04523 |
|  |  | 30 | GO:0044421 | extracellular region part | 4578 | 15 | 9.7 | 0.04528 |
|  |  | 31 | GO:0070062 | extracellular exosome | 3105 | 11 | 6.58 | 0.05545 |
|  |  | 32 | GO:1903561 | extracellular vesicle | 3127 | 11 | 6.63 | 0.05787 |
|  |  | 33 | GO:0043230 | extracellular organelle | 3129 | 11 | 6.63 | 0.05809 |
|  |  | 34 | GO:0005775 | vacuolar lumen | 203 | 2 | 0.43 | 0.06889 |
|  |  | 35 | GO:0001891 | phagocytic cup | 35 | 1 | 0.07 | 0.07163 |
|  |  | 36 | GO:0002102 | podosome | 36 | 1 | 0.08 | 0.0736 |
|  |  | 37 | GO:0033017 | sarcoplasmic reticulum membrane | 39 | 1 | 0.08 | 0.07949 |
|  |  | 38 | GO:0043198 | dendritic shaft | 40 | 1 | 0.08 | 0.08145 |
|  |  | 39 | GO:0099513 | polymeric cytoskeletal fiber | 801 | 4 | 1.7 | 0.08879 |
|  |  | 40 | GO:0046658 | anchored component of plasma membrane | 49 | 1 | 0.1 | 0.09886 |
|  |  | 41 | GO:0005615 | extracellular space | 4339 | 13 | 9.2 | 0.11264 |
|  |  | 42 | GO:0005871 | kinesin complex | 62 | 1 | 0.13 | 0.12344 |
|  |  | 43 | GO:0016529 | sarcoplasmic reticulum | 62 | 1 | 0.13 | 0.12344 |
|  |  | 44 | GO:0009897 | external side of plasma membrane | 295 | 2 | 0.63 | 0.12925 |
|  |  | 45 | GO:0009986 | cell surface | 936 | 4 | 1.98 | 0.13553 |
|  |  | 46 | GO:0031982 | vesicle | 4920 | 14 | 10.43 | 0.13865 |
|  |  | 47 | GO:0016528 | sarcoplasm | 71 | 1 | 0.15 | 0.14008 |
|  |  | 48 | GO:0045121 | membrane raft | 336 | 2 | 0.71 | 0.15911 |
|  |  | 49 | GO:0098857 | membrane microdomain | 337 | 2 | 0.71 | 0.15986 |
|  |  | 50 | GO:0030864 | cortical actin cytoskeleton | 83 | 1 | 0.18 | 0.16177 |
|  | MF | 1 | GO:0005178 | integrin binding | 129 | 4 | 0.29 | 0.00019 |
|  |  | 2 | GO:0050839 | cell adhesion molecule binding | 541 | 6 | 1.2 | 0.00115 |
|  |  | 3 | GO:0016316 | phosphatidylinositol-3,4-bisphosphate 4-... | 2 | 1 | 0 | 0.00442 |
|  |  | 4 | GO:0017089 | glycolipid transporter activity | 2 | 1 | 0 | 0.00442 |
|  |  | 5 | GO:0017161 | inositol-1,3,4-trisphosphate 4-phosphata... | 2 | 1 | 0 | 0.00442 |
|  |  | 6 | GO:0052828 | inositol-3,4-bisphosphate 4-phosphatase ... | 2 | 1 | 0 | 0.00442 |
|  |  | 7 | GO:0001605 | adrenomedullin receptor activity | 3 | 1 | 0.01 | 0.00662 |
|  |  | 8 | GO:0016312 | inositol bisphosphate phosphatase activi... | 4 | 1 | 0.01 | 0.00882 |
|  |  | 9 | GO:0034597 | phosphatidylinositol-4,5-bisphosphate 4-... | 4 | 1 | 0.01 | 0.00882 |
|  |  | 10 | GO:0097643 | amylin receptor activity | 4 | 1 | 0.01 | 0.00882 |
|  |  | 11 | GO:0048495 | Roundabout binding | 5 | 1 | 0.01 | 0.01101 |
|  |  | 12 | GO:0106017 | phosphatidylinositol-3,4-bisphosphate ph... | 5 | 1 | 0.01 | 0.01101 |
|  |  | 13 | GO:0097642 | calcitonin family receptor activity | 6 | 1 | 0.01 | 0.0132 |
|  |  | 14 | GO:0120013 | intermembrane lipid transfer activity | 7 | 1 | 0.02 | 0.01538 |
|  |  | 15 | GO:0005102 | signaling receptor binding | 1810 | 9 | 4 | 0.01586 |
|  |  | 16 | GO:0038036 | sphingosine-1-phosphate receptor activit... | 8 | 1 | 0.02 | 0.01756 |
|  |  | 17 | GO:0005201 | extracellular matrix structural constitu... | 95 | 2 | 0.21 | 0.01873 |
|  |  | 18 | GO:0004860 | protein kinase inhibitor activity | 102 | 2 | 0.23 | 0.0214 |
|  |  | 19 | GO:0034596 | phosphatidylinositol phosphate 4-phospha... | 10 | 1 | 0.02 | 0.0219 |
|  |  | 20 | GO:0019210 | kinase inhibitor activity | 107 | 2 | 0.24 | 0.0234 |
|  |  | 21 | GO:0001849 | complement component C1q binding | 11 | 1 | 0.02 | 0.02407 |
|  |  | 22 | GO:0005509 | calcium ion binding | 752 | 5 | 1.66 | 0.02443 |
|  |  | 23 | GO:0046030 | inositol trisphosphate phosphatase activ... | 12 | 1 | 0.03 | 0.02623 |
|  |  | 24 | GO:0106019 | phosphatidylinositol-4,5-bisphosphate ph... | 13 | 1 | 0.03 | 0.02838 |
|  |  | 25 | GO:0005388 | calcium-transporting ATPase activity | 14 | 1 | 0.03 | 0.03053 |
|  |  | 26 | GO:0047485 | protein N-terminus binding | 127 | 2 | 0.28 | 0.03211 |
|  |  | 27 | GO:0045125 | bioactive lipid receptor activity | 15 | 1 | 0.03 | 0.03268 |
|  |  | 28 | GO:0001846 | opsonin binding | 17 | 1 | 0.04 | 0.03695 |
|  |  | 29 | GO:0045236 | CXCR chemokine receptor binding | 17 | 1 | 0.04 | 0.03695 |
|  |  | 30 | GO:0051861 | glycolipid binding | 19 | 1 | 0.04 | 0.04121 |
|  |  | 31 | GO:0005319 | lipid transporter activity | 148 | 2 | 0.33 | 0.04241 |
|  |  | 32 | GO:0008553 | proton-exporting ATPase activity, phosph... | 21 | 1 | 0.05 | 0.04545 |
|  |  | 33 | GO:0050750 | low-density lipoprotein particle recepto... | 21 | 1 | 0.05 | 0.04545 |
|  |  | 34 | GO:0015631 | tubulin binding | 366 | 3 | 0.81 | 0.04694 |
|  |  | 35 | GO:0052745 | inositol phosphate phosphatase activity | 22 | 1 | 0.05 | 0.04757 |
|  |  | 36 | GO:0043492 | ATPase activity, coupled to movement of ... | 159 | 2 | 0.35 | 0.04824 |
|  |  | 37 | GO:0004012 | phospholipid-translocating ATPase activi... | 23 | 1 | 0.05 | 0.04968 |
|  |  | 38 | GO:0005112 | Notch binding | 23 | 1 | 0.05 | 0.04968 |
|  |  | 39 | GO:0042813 | Wnt-activated receptor activity | 23 | 1 | 0.05 | 0.04968 |
|  |  | 40 | GO:0070577 | lysine-acetylated histone binding | 24 | 1 | 0.05 | 0.05178 |
|  |  | 41 | GO:0140033 | acetylation-dependent protein binding | 24 | 1 | 0.05 | 0.05178 |
|  |  | 42 | GO:0070412 | R-SMAD binding | 25 | 1 | 0.06 | 0.05388 |
|  |  | 43 | GO:0070325 | lipoprotein particle receptor binding | 26 | 1 | 0.06 | 0.05597 |
|  |  | 44 | GO:0005515 | protein binding | 13002 | 34 | 28.76 | 0.05802 |
|  |  | 45 | GO:0032452 | histone demethylase activity | 28 | 1 | 0.06 | 0.06015 |
|  |  | 46 | GO:0034593 | phosphatidylinositol bisphosphate phosph... | 29 | 1 | 0.06 | 0.06223 |
|  |  | 47 | GO:0017147 | Wnt-protein binding | 32 | 1 | 0.07 | 0.06845 |
|  |  | 48 | GO:0050699 | WW domain binding | 32 | 1 | 0.07 | 0.06845 |
|  |  | 49 | GO:0001530 | lipopolysaccharide binding | 33 | 1 | 0.07 | 0.07051 |
|  |  | 50 | GO:0001848 | complement binding | 33 | 1 | 0.07 | 0.07051 |
| Green | BP | 1 | GO:0003148 | outflow tract septum morphogenesis | 25 | 2 | 0.03 | 0.00046 |
|  |  | 2 | GO:0003206 | cardiac chamber morphogenesis | 125 | 3 | 0.16 | 0.00052 |
|  |  | 3 | GO:0072001 | renal system development | 300 | 4 | 0.38 | 0.00053 |
|  |  | 4 | GO:0001655 | urogenital system development | 337 | 4 | 0.43 | 0.00081 |
|  |  | 5 | GO:0048565 | digestive tract development | 151 | 3 | 0.19 | 0.00091 |
|  |  | 6 | GO:0003205 | cardiac chamber development | 160 | 3 | 0.2 | 0.00107 |
|  |  | 7 | GO:0055123 | digestive system development | 163 | 3 | 0.21 | 0.00113 |
|  |  | 8 | GO:0045769 | negative regulation of asymmetric cell d... | 1 | 1 | 0 | 0.00127 |
|  |  | 9 | GO:0060177 | regulation of angiotensin metabolic proc... | 1 | 1 | 0 | 0.00127 |
|  |  | 10 | GO:1903597 | negative regulation of gap junction asse... | 1 | 1 | 0 | 0.00127 |
|  |  | 11 | GO:0060976 | coronary vasculature development | 47 | 2 | 0.06 | 0.00163 |
|  |  | 12 | GO:1905209 | positive regulation of cardiocyte differ... | 48 | 2 | 0.06 | 0.0017 |
|  |  | 13 | GO:0035904 | aorta development | 52 | 2 | 0.07 | 0.00199 |
|  |  | 14 | GO:0019320 | hexose catabolic process | 55 | 2 | 0.07 | 0.00222 |
|  |  | 15 | GO:0033627 | cell adhesion mediated by integrin | 58 | 2 | 0.07 | 0.00247 |
|  |  | 16 | GO:0045596 | negative regulation of cell differentiat... | 769 | 5 | 0.98 | 0.00252 |
|  |  | 17 | GO:0007493 | endodermal cell fate determination | 2 | 1 | 0 | 0.00255 |
|  |  | 18 | GO:0010983 | positive regulation of high-density lipo... | 2 | 1 | 0 | 0.00255 |
|  |  | 19 | GO:0032912 | negative regulation of transforming grow... | 2 | 1 | 0 | 0.00255 |
|  |  | 20 | GO:0072334 | UDP-galactose transmembrane transport | 2 | 1 | 0 | 0.00255 |
|  |  | 21 | GO:1903849 | positive regulation of aorta morphogenes... | 2 | 1 | 0 | 0.00255 |
|  |  | 22 | GO:2000170 | positive regulation of peptidyl-cysteine... | 2 | 1 | 0 | 0.00255 |
|  |  | 23 | GO:0072091 | regulation of stem cell proliferation | 63 | 2 | 0.08 | 0.0029 |
|  |  | 24 | GO:0046365 | monosaccharide catabolic process | 65 | 2 | 0.08 | 0.00309 |
|  |  | 25 | GO:0055025 | positive regulation of cardiac muscle ti... | 67 | 2 | 0.09 | 0.00327 |
|  |  | 26 | GO:0048738 | cardiac muscle tissue development | 237 | 3 | 0.3 | 0.00329 |
|  |  | 27 | GO:0060411 | cardiac septum morphogenesis | 70 | 2 | 0.09 | 0.00357 |
|  |  | 28 | GO:1905207 | regulation of cardiocyte differentiation | 70 | 2 | 0.09 | 0.00357 |
|  |  | 29 | GO:0002376 | immune system process | 3719 | 11 | 4.74 | 0.00358 |
|  |  | 30 | GO:0003007 | heart morphogenesis | 249 | 3 | 0.32 | 0.00378 |
|  |  | 31 | GO:0002019 | regulation of renal output by angiotensi... | 3 | 1 | 0 | 0.00382 |
|  |  | 32 | GO:0007352 | zygotic specification of dorsal/ventral ... | 3 | 1 | 0 | 0.00382 |
|  |  | 33 | GO:0010982 | regulation of high-density lipoprotein p... | 3 | 1 | 0 | 0.00382 |
|  |  | 34 | GO:0110024 | positive regulation of cardiac muscle my... | 3 | 1 | 0 | 0.00382 |
|  |  | 35 | GO:0034329 | cell junction assembly | 250 | 3 | 0.32 | 0.00382 |
|  |  | 36 | GO:0003151 | outflow tract morphogenesis | 75 | 2 | 0.1 | 0.00408 |
|  |  | 37 | GO:0043627 | response to estrogen | 75 | 2 | 0.1 | 0.00408 |
|  |  | 38 | GO:0051179 | localization | 7075 | 16 | 9.01 | 0.00412 |
|  |  | 39 | GO:0051241 | negative regulation of multicellular org... | 1283 | 6 | 1.63 | 0.00466 |
|  |  | 40 | GO:0001951 | intestinal D-glucose absorption | 4 | 1 | 0.01 | 0.00509 |
|  |  | 41 | GO:0002159 | desmosome assembly | 4 | 1 | 0.01 | 0.00509 |
|  |  | 42 | GO:0010716 | negative regulation of extracellular mat... | 4 | 1 | 0.01 | 0.00509 |
|  |  | 43 | GO:0032911 | negative regulation of transforming grow... | 4 | 1 | 0.01 | 0.00509 |
|  |  | 44 | GO:0036343 | psychomotor behavior | 4 | 1 | 0.01 | 0.00509 |
|  |  | 45 | GO:0060486 | Clara cell differentiation | 4 | 1 | 0.01 | 0.00509 |
|  |  | 46 | GO:1903596 | regulation of gap junction assembly | 4 | 1 | 0.01 | 0.00509 |
|  |  | 47 | GO:1905653 | positive regulation of artery morphogene... | 4 | 1 | 0.01 | 0.00509 |
|  |  | 48 | GO:0001822 | kidney development | 284 | 3 | 0.36 | 0.00545 |
|  |  | 49 | GO:0007507 | heart development | 575 | 4 | 0.73 | 0.00566 |
|  |  | 50 | GO:0060840 | artery development | 91 | 2 | 0.12 | 0.00595 |
|  | CC | 1 | GO:0009986 | cell surface | 936 | 6 | 1.17 | 0.00088 |
|  |  | 2 | GO:0034676 | integrin alpha6-beta4 complex | 1 | 1 | 0 | 0.00125 |
|  |  | 3 | GO:0005911 | cell-cell junction | 479 | 4 | 0.6 | 0.0028 |
|  |  | 4 | GO:0097129 | cyclin D2-CDK4 complex | 3 | 1 | 0 | 0.00375 |
|  |  | 5 | GO:0012505 | endomembrane system | 5028 | 13 | 6.3 | 0.00387 |
|  |  | 6 | GO:0000015 | phosphopyruvate hydratase complex | 4 | 1 | 0.01 | 0.005 |
|  |  | 7 | GO:0005912 | adherens junction | 604 | 4 | 0.76 | 0.00638 |
|  |  | 8 | GO:0070161 | anchoring junction | 627 | 4 | 0.79 | 0.00726 |
|  |  | 9 | GO:0016020 | membrane | 10723 | 20 | 13.43 | 0.00748 |
|  |  | 10 | GO:0071438 | invadopodium membrane | 6 | 1 | 0.01 | 0.00749 |
|  |  | 11 | GO:0016324 | apical plasma membrane | 335 | 3 | 0.42 | 0.00823 |
|  |  | 12 | GO:0030175 | filopodium | 110 | 2 | 0.14 | 0.00832 |
|  |  | 13 | GO:0030056 | hemidesmosome | 7 | 1 | 0.01 | 0.00874 |
|  |  | 14 | GO:0046581 | intercellular canaliculus | 7 | 1 | 0.01 | 0.00874 |
|  |  | 15 | GO:0036449 | microtubule minus-end | 8 | 1 | 0.01 | 0.00998 |
|  |  | 16 | GO:0072687 | meiotic spindle | 9 | 1 | 0.01 | 0.01122 |
|  |  | 17 | GO:0045177 | apical part of cell | 404 | 3 | 0.51 | 0.01365 |
|  |  | 18 | GO:0071944 | cell periphery | 6532 | 14 | 8.18 | 0.01469 |
|  |  | 19 | GO:0005925 | focal adhesion | 451 | 3 | 0.56 | 0.01828 |
|  |  | 20 | GO:0005924 | cell-substrate adherens junction | 454 | 3 | 0.57 | 0.01861 |
|  |  | 21 | GO:0030055 | cell-substrate junction | 459 | 3 | 0.57 | 0.01915 |
|  |  | 22 | GO:0071437 | invadopodium | 16 | 1 | 0.02 | 0.01986 |
|  |  | 23 | GO:0005576 | extracellular region | 5443 | 12 | 6.82 | 0.02241 |
|  |  | 24 | GO:0001931 | uropod | 20 | 1 | 0.03 | 0.02477 |
|  |  | 25 | GO:0031254 | cell trailing edge | 20 | 1 | 0.03 | 0.02477 |
|  |  | 26 | GO:0030027 | lamellipodium | 204 | 2 | 0.26 | 0.02676 |
|  |  | 27 | GO:0031258 | lamellipodium membrane | 22 | 1 | 0.03 | 0.02721 |
|  |  | 28 | GO:0030054 | cell junction | 1398 | 5 | 1.75 | 0.02764 |
|  |  | 29 | GO:0098858 | actin-based cell projection | 218 | 2 | 0.27 | 0.03024 |
|  |  | 30 | GO:0070062 | extracellular exosome | 3105 | 8 | 3.89 | 0.03151 |
|  |  | 31 | GO:1990752 | microtubule end | 26 | 1 | 0.03 | 0.03208 |
|  |  | 32 | GO:0005886 | plasma membrane | 6420 | 13 | 8.04 | 0.03255 |
|  |  | 33 | GO:1903561 | extracellular vesicle | 3127 | 8 | 3.92 | 0.03273 |
|  |  | 34 | GO:0043230 | extracellular organelle | 3129 | 8 | 3.92 | 0.03284 |
|  |  | 35 | GO:0097431 | mitotic spindle pole | 27 | 1 | 0.03 | 0.03329 |
|  |  | 36 | GO:0030057 | desmosome | 30 | 1 | 0.04 | 0.03693 |
|  |  | 37 | GO:0008305 | integrin complex | 32 | 1 | 0.04 | 0.03934 |
|  |  | 38 | GO:0098636 | protein complex involved in cell adhesio... | 35 | 1 | 0.04 | 0.04295 |
|  |  | 39 | GO:0044421 | extracellular region part | 4578 | 10 | 5.73 | 0.04329 |
|  |  | 40 | GO:0031941 | filamentous actin | 38 | 1 | 0.05 | 0.04655 |
|  |  | 41 | GO:0000307 | cyclin-dependent protein kinase holoenzy... | 42 | 1 | 0.05 | 0.05133 |
|  |  | 42 | GO:0000788 | nuclear nucleosome | 42 | 1 | 0.05 | 0.05133 |
|  |  | 43 | GO:0009925 | basal plasma membrane | 42 | 1 | 0.05 | 0.05133 |
|  |  | 44 | GO:0009897 | external side of plasma membrane | 295 | 2 | 0.37 | 0.0523 |
|  |  | 45 | GO:0030173 | integral component of Golgi membrane | 44 | 1 | 0.06 | 0.05371 |
|  |  | 46 | GO:0098590 | plasma membrane region | 1170 | 4 | 1.47 | 0.05583 |
|  |  | 47 | GO:0001917 | photoreceptor inner segment | 47 | 1 | 0.06 | 0.05727 |
|  |  | 48 | GO:0031228 | intrinsic component of Golgi membrane | 49 | 1 | 0.06 | 0.05963 |
|  |  | 49 | GO:0014704 | intercalated disc | 50 | 1 | 0.06 | 0.06081 |
|  |  | 50 | GO:0005794 | Golgi apparatus | 1758 | 5 | 2.2 | 0.06389 |
|  | MF | 1 | GO:0019899 | enzyme binding | 2527 | 11 | 3.25 | 0.00013 |
|  |  | 2 | GO:0008238 | exopeptidase activity | 111 | 3 | 0.14 | 0.00038 |
|  |  | 3 | GO:0004180 | carboxypeptidase activity | 45 | 2 | 0.06 | 0.00152 |
|  |  | 4 | GO:0019901 | protein kinase binding | 708 | 5 | 0.91 | 0.00183 |
|  |  | 5 | GO:0005459 | UDP-galactose transmembrane transporter ... | 2 | 1 | 0 | 0.00257 |
|  |  | 6 | GO:0008241 | peptidyl-dipeptidase activity | 2 | 1 | 0 | 0.00257 |
|  |  | 7 | GO:0030617 | transforming growth factor beta receptor... | 2 | 1 | 0 | 0.00257 |
|  |  | 8 | GO:0031711 | bradykinin receptor binding | 2 | 1 | 0 | 0.00257 |
|  |  | 9 | GO:0052810 | 1-phosphatidylinositol-5-kinase activity | 2 | 1 | 0 | 0.00257 |
|  |  | 10 | GO:0070698 | type I activin receptor binding | 2 | 1 | 0 | 0.00257 |
|  |  | 11 | GO:0008235 | metalloexopeptidase activity | 60 | 2 | 0.08 | 0.00268 |
|  |  | 12 | GO:0019900 | kinase binding | 788 | 5 | 1.01 | 0.00292 |
|  |  | 13 | GO:0008240 | tripeptidyl-peptidase activity | 3 | 1 | 0 | 0.00385 |
|  |  | 14 | GO:0052812 | phosphatidylinositol-3,4-bisphosphate 5-... | 3 | 1 | 0 | 0.00385 |
|  |  | 15 | GO:0070573 | metallodipeptidase activity | 3 | 1 | 0 | 0.00385 |
|  |  | 16 | GO:0000285 | 1-phosphatidylinositol-3-phosphate 5-kin... | 4 | 1 | 0.01 | 0.00513 |
|  |  | 17 | GO:0004634 | phosphopyruvate hydratase activity | 4 | 1 | 0.01 | 0.00513 |
|  |  | 18 | GO:0001618 | virus receptor activity | 91 | 2 | 0.12 | 0.00606 |
|  |  | 19 | GO:0104005 | hijacked molecular function | 91 | 2 | 0.12 | 0.00606 |
|  |  | 20 | GO:0038132 | neuregulin binding | 5 | 1 | 0.01 | 0.00641 |
|  |  | 21 | GO:0086083 | cell adhesive protein binding involved i... | 5 | 1 | 0.01 | 0.00641 |
|  |  | 22 | GO:0005412 | glucose:sodium symporter activity | 6 | 1 | 0.01 | 0.00769 |
|  |  | 23 | GO:0052811 | 1-phosphatidylinositol-3-phosphate 4-kin... | 6 | 1 | 0.01 | 0.00769 |
|  |  | 24 | GO:0004465 | lipoprotein lipase activity | 8 | 1 | 0.01 | 0.01024 |
|  |  | 25 | GO:0016308 | 1-phosphatidylinositol-4-phosphate 5-kin... | 8 | 1 | 0.01 | 0.01024 |
|  |  | 26 | GO:0046920 | alpha-(1->3)-fucosyltransferase activity | 8 | 1 | 0.01 | 0.01024 |
|  |  | 27 | GO:0070697 | activin receptor binding | 8 | 1 | 0.01 | 0.01024 |
|  |  | 28 | GO:0071933 | Arp2/3 complex binding | 8 | 1 | 0.01 | 0.01024 |
|  |  | 29 | GO:0031404 | chloride ion binding | 9 | 1 | 0.01 | 0.01152 |
|  |  | 30 | GO:0034713 | type I transforming growth factor beta r... | 9 | 1 | 0.01 | 0.01152 |
|  |  | 31 | GO:0005072 | transforming growth factor beta receptor... | 10 | 1 | 0.01 | 0.01279 |
|  |  | 32 | GO:0015165 | pyrimidine nucleotide-sugar transmembran... | 10 | 1 | 0.01 | 0.01279 |
|  |  | 33 | GO:0019911 | structural constituent of myelin sheath | 10 | 1 | 0.01 | 0.01279 |
|  |  | 34 | GO:0086080 | protein binding involved in heterotypic ... | 10 | 1 | 0.01 | 0.01279 |
|  |  | 35 | GO:0008970 | phosphatidylcholine 1-acylhydrolase acti... | 11 | 1 | 0.01 | 0.01406 |
|  |  | 36 | GO:0045294 | alpha-catenin binding | 11 | 1 | 0.01 | 0.01406 |
|  |  | 37 | GO:0005338 | nucleotide-sugar transmembrane transport... | 12 | 1 | 0.02 | 0.01533 |
|  |  | 38 | GO:0008239 | dipeptidyl-peptidase activity | 12 | 1 | 0.02 | 0.01533 |
|  |  | 39 | GO:0070411 | I-SMAD binding | 12 | 1 | 0.02 | 0.01533 |
|  |  | 40 | GO:0008417 | fucosyltransferase activity | 13 | 1 | 0.02 | 0.01659 |
|  |  | 41 | GO:0031994 | insulin-like growth factor I binding | 13 | 1 | 0.02 | 0.01659 |
|  |  | 42 | GO:0070410 | co-SMAD binding | 13 | 1 | 0.02 | 0.01659 |
|  |  | 43 | GO:0005198 | structural molecule activity | 809 | 4 | 1.04 | 0.01875 |
|  |  | 44 | GO:0005095 | GTPase inhibitor activity | 15 | 1 | 0.02 | 0.01912 |
|  |  | 45 | GO:0016805 | dipeptidase activity | 16 | 1 | 0.02 | 0.02038 |
|  |  | 46 | GO:0019215 | intermediate filament binding | 16 | 1 | 0.02 | 0.02038 |
|  |  | 47 | GO:0016307 | phosphatidylinositol phosphate kinase ac... | 18 | 1 | 0.02 | 0.0229 |
|  |  | 48 | GO:0005402 | carbohydrate:cation symporter activity | 20 | 1 | 0.03 | 0.02542 |
|  |  | 49 | GO:0070696 | transmembrane receptor protein serine/th... | 20 | 1 | 0.03 | 0.02542 |
|  |  | 50 | GO:0008237 | metallopeptidase activity | 200 | 2 | 0.26 | 0.02704 |
| Turquoise | BP | 1 | GO:0045935 | positive regulation of nucleobase-contai... | 1870 | 25 | 8.29 | 3.20E-07 |
|  |  | 2 | GO:0051254 | positive regulation of RNA metabolic pro... | 1638 | 23 | 7.26 | 4.60E-07 |
|  |  | 3 | GO:0045893 | positive regulation of transcription, DN... | 1554 | 22 | 6.89 | 7.70E-07 |
|  |  | 4 | GO:1903508 | positive regulation of nucleic acid-temp... | 1554 | 22 | 6.89 | 7.70E-07 |
|  |  | 5 | GO:1902680 | positive regulation of RNA biosynthetic ... | 1555 | 22 | 6.89 | 7.70E-07 |
|  |  | 6 | GO:0000122 | negative regulation of transcription by ... | 863 | 16 | 3.83 | 1.10E-06 |
|  |  | 7 | GO:0045944 | positive regulation of transcription by ... | 1216 | 19 | 5.39 | 1.20E-06 |
|  |  | 8 | GO:0031325 | positive regulation of cellular metaboli... | 3351 | 33 | 14.86 | 2.80E-06 |
|  |  | 9 | GO:0010557 | positive regulation of macromolecule bio... | 1826 | 23 | 8.1 | 3.00E-06 |
|  |  | 10 | GO:0051173 | positive regulation of nitrogen compound... | 3234 | 32 | 14.34 | 3.90E-06 |
|  |  | 11 | GO:0010628 | positive regulation of gene expression | 2014 | 24 | 8.93 | 4.60E-06 |
|  |  | 12 | GO:0051172 | negative regulation of nitrogen compound... | 2839 | 29 | 12.59 | 7.30E-06 |
|  |  | 13 | GO:0031324 | negative regulation of cellular metaboli... | 3003 | 30 | 13.31 | 7.40E-06 |
|  |  | 14 | GO:0031328 | positive regulation of cellular biosynth... | 1951 | 23 | 8.65 | 9.20E-06 |
|  |  | 15 | GO:0045892 | negative regulation of transcription, DN... | 1283 | 18 | 5.69 | 1.10E-05 |
|  |  | 16 | GO:0009891 | positive regulation of biosynthetic proc... | 1980 | 23 | 8.78 | 1.20E-05 |
|  |  | 17 | GO:0009893 | positive regulation of metabolic process | 3615 | 33 | 16.03 | 1.50E-05 |
|  |  | 18 | GO:0048522 | positive regulation of cellular process | 5680 | 44 | 25.18 | 1.70E-05 |
|  |  | 19 | GO:1903507 | negative regulation of nucleic acid-temp... | 1331 | 18 | 5.9 | 1.80E-05 |
|  |  | 20 | GO:1902679 | negative regulation of RNA biosynthetic ... | 1333 | 18 | 5.91 | 1.80E-05 |
|  |  | 21 | GO:0048627 | myoblast development | 2 | 2 | 0.01 | 1.90E-05 |
|  |  | 22 | GO:0042594 | response to starvation | 188 | 7 | 0.83 | 2.00E-05 |
|  |  | 23 | GO:2000113 | negative regulation of cellular macromol... | 1760 | 21 | 7.8 | 2.10E-05 |
|  |  | 24 | GO:0048523 | negative regulation of cellular process | 5330 | 42 | 23.63 | 2.10E-05 |
|  |  | 25 | GO:0031327 | negative regulation of cellular biosynth... | 1929 | 22 | 8.55 | 2.50E-05 |
|  |  | 26 | GO:0010604 | positive regulation of macromolecule met... | 3379 | 31 | 14.98 | 2.80E-05 |
|  |  | 27 | GO:0009890 | negative regulation of biosynthetic proc... | 1949 | 22 | 8.64 | 3.00E-05 |
|  |  | 28 | GO:0006357 | regulation of transcription by RNA polym... | 2406 | 25 | 10.67 | 3.00E-05 |
|  |  | 29 | GO:0048518 | positive regulation of biological proces... | 6433 | 47 | 28.52 | 3.40E-05 |
|  |  | 30 | GO:0006366 | transcription by RNA polymerase II | 2599 | 26 | 11.52 | 3.80E-05 |
|  |  | 31 | GO:0010558 | negative regulation of macromolecule bio... | 1838 | 21 | 8.15 | 4.00E-05 |
|  |  | 32 | GO:0051253 | negative regulation of RNA metabolic pro... | 1415 | 18 | 6.27 | 4.00E-05 |
|  |  | 33 | GO:0009605 | response to external stimulus | 2452 | 25 | 10.87 | 4.10E-05 |
|  |  | 34 | GO:0045934 | negative regulation of nucleobase-contai... | 1575 | 19 | 6.98 | 4.90E-05 |
|  |  | 35 | GO:0009888 | tissue development | 2175 | 23 | 9.64 | 5.30E-05 |
|  |  | 36 | GO:0010605 | negative regulation of macromolecule met... | 3147 | 29 | 13.95 | 5.30E-05 |
|  |  | 37 | GO:0051726 | regulation of cell cycle | 1343 | 17 | 5.95 | 7.30E-05 |
|  |  | 38 | GO:0042752 | regulation of circadian rhythm | 99 | 5 | 0.44 | 7.90E-05 |
|  |  | 39 | GO:0009892 | negative regulation of metabolic process | 3408 | 30 | 15.11 | 8.90E-05 |
|  |  | 40 | GO:0043153 | entrainment of circadian clock by photop... | 20 | 3 | 0.09 | 9.10E-05 |
|  |  | 41 | GO:0048545 | response to steroid hormone | 425 | 9 | 1.88 | 0.00011 |
|  |  | 42 | GO:0008219 | cell death | 2453 | 24 | 10.88 | 0.00012 |
|  |  | 43 | GO:0008283 | cell proliferation | 2299 | 23 | 10.19 | 0.00012 |
|  |  | 44 | GO:0008544 | epidermis development | 536 | 10 | 2.38 | 0.00013 |
|  |  | 45 | GO:0048519 | negative regulation of biological proces... | 5929 | 43 | 26.29 | 0.00013 |
|  |  | 46 | GO:0012501 | programmed cell death | 2317 | 23 | 10.27 | 0.00014 |
|  |  | 47 | GO:0048856 | anatomical structure development | 6364 | 45 | 28.22 | 0.00015 |
|  |  | 48 | GO:0042127 | regulation of cell proliferation | 1863 | 20 | 8.26 | 0.00015 |
|  |  | 49 | GO:0033554 | cellular response to stress | 2176 | 22 | 9.65 | 0.00016 |
|  |  | 50 | GO:0009648 | photoperiodism | 24 | 3 | 0.11 | 0.00016 |
|  | CC | 1 | GO:0005634 | nucleus | 7961 | 53 | 33.37 | 1.60E-05 |
|  |  | 2 | GO:0043226 | organelle | 15040 | 78 | 63.03 | 7.80E-05 |
|  |  | 3 | GO:0043227 | membrane-bounded organelle | 13959 | 74 | 58.5 | 0.00013 |
|  |  | 4 | GO:0043229 | intracellular organelle | 13979 | 73 | 58.59 | 0.00039 |
|  |  | 5 | GO:0043231 | intracellular membrane-bounded organelle | 12050 | 66 | 50.5 | 0.00039 |
|  |  | 6 | GO:0044424 | intracellular part | 15901 | 79 | 66.64 | 0.00053 |
|  |  | 7 | GO:0005667 | transcription factor complex | 373 | 7 | 1.56 | 0.00097 |
|  |  | 8 | GO:0005622 | intracellular | 16208 | 79 | 67.93 | 0.00149 |
|  |  | 9 | GO:0005829 | cytosol | 5508 | 35 | 23.08 | 0.00368 |
|  |  | 10 | GO:0005912 | adherens junction | 604 | 8 | 2.53 | 0.00382 |
|  |  | 11 | GO:0098745 | Dcp1-Dcp2 complex | 1 | 1 | 0 | 0.00419 |
|  |  | 12 | GO:0070161 | anchoring junction | 627 | 8 | 2.63 | 0.00477 |
|  |  | 13 | GO:0043228 | non-membrane-bounded organelle | 4552 | 30 | 19.08 | 0.00483 |
|  |  | 14 | GO:0043232 | intracellular non-membrane-bounded organ... | 4552 | 30 | 19.08 | 0.00483 |
|  |  | 15 | GO:0031981 | nuclear lumen | 4533 | 29 | 19 | 0.00882 |
|  |  | 16 | GO:0000786 | nucleosome | 108 | 3 | 0.45 | 0.01058 |
|  |  | 17 | GO:0044464 | cell part | 18844 | 85 | 78.98 | 0.0106 |
|  |  | 18 | GO:0044798 | nuclear transcription factor complex | 205 | 4 | 0.86 | 0.01087 |
|  |  | 19 | GO:0005623 | cell | 18876 | 85 | 79.11 | 0.01189 |
|  |  | 20 | GO:0020016 | ciliary pocket | 3 | 1 | 0.01 | 0.01252 |
|  |  | 21 | GO:0020018 | ciliary pocket membrane | 3 | 1 | 0.01 | 0.01252 |
|  |  | 22 | GO:0035189 | Rb-E2F complex | 3 | 1 | 0.01 | 0.01252 |
|  |  | 23 | GO:1905286 | serine-type peptidase complex | 3 | 1 | 0.01 | 0.01252 |
|  |  | 24 | GO:0044815 | DNA packaging complex | 115 | 3 | 0.48 | 0.01253 |
|  |  | 25 | GO:0000785 | chromatin | 596 | 7 | 2.5 | 0.01256 |
|  |  | 26 | GO:0005737 | cytoplasm | 12796 | 64 | 53.63 | 0.01309 |
|  |  | 27 | GO:0044449 | contractile fiber part | 224 | 4 | 0.94 | 0.01464 |
|  |  | 28 | GO:0044428 | nuclear part | 4922 | 30 | 20.63 | 0.01501 |
|  |  | 29 | GO:0030016 | myofibril | 229 | 4 | 0.96 | 0.01575 |
|  |  | 30 | GO:0010494 | cytoplasmic stress granule | 46 | 2 | 0.19 | 0.01595 |
|  |  | 31 | GO:0035517 | PR-DUB complex | 4 | 1 | 0.02 | 0.01666 |
|  |  | 32 | GO:0072558 | NLRP1 inflammasome complex | 4 | 1 | 0.02 | 0.01666 |
|  |  | 33 | GO:0005913 | cell-cell adherens junction | 130 | 3 | 0.54 | 0.01737 |
|  |  | 34 | GO:0043292 | contractile fiber | 241 | 4 | 1.01 | 0.01863 |
|  |  | 35 | GO:0033553 | rDNA heterochromatin | 5 | 1 | 0.02 | 0.02078 |
|  |  | 36 | GO:0035976 | transcription factor AP-1 complex | 5 | 1 | 0.02 | 0.02078 |
|  |  | 37 | GO:0098983 | symmetric, GABA-ergic, inhibitory synaps... | 5 | 1 | 0.02 | 0.02078 |
|  |  | 38 | GO:0019005 | SCF ubiquitin ligase complex | 58 | 2 | 0.24 | 0.02466 |
|  |  | 39 | GO:0098978 | glutamatergic synapse | 6 | 1 | 0.03 | 0.02489 |
|  |  | 40 | GO:0098982 | GABA-ergic synapse | 6 | 1 | 0.03 | 0.02489 |
|  |  | 41 | GO:0098985 | asymmetric, glutamatergic, excitatory sy... | 6 | 1 | 0.03 | 0.02489 |
|  |  | 42 | GO:0031974 | membrane-enclosed lumen | 5776 | 33 | 24.21 | 0.02586 |
|  |  | 43 | GO:0043233 | organelle lumen | 5776 | 33 | 24.21 | 0.02586 |
|  |  | 44 | GO:0070013 | intracellular organelle lumen | 5776 | 33 | 24.21 | 0.02586 |
|  |  | 45 | GO:0044446 | intracellular organelle part | 10167 | 52 | 42.61 | 0.02786 |
|  |  | 46 | GO:0097169 | AIM2 inflammasome complex | 7 | 1 | 0.03 | 0.02898 |
|  |  | 47 | GO:0099512 | supramolecular fiber | 1046 | 9 | 4.38 | 0.03095 |
|  |  | 48 | GO:0008287 | protein serine/threonine phosphatase com... | 66 | 2 | 0.28 | 0.03132 |
|  |  | 49 | GO:1903293 | phosphatase complex | 66 | 2 | 0.28 | 0.03132 |
|  |  | 50 | GO:0030054 | cell junction | 1398 | 11 | 5.86 | 0.0315 |
|  | MF | 1 | GO:0000982 | transcription factor activity, RNA polym... | 424 | 14 | 1.88 | 4.80E-09 |
|  |  | 2 | GO:0000978 | RNA polymerase II proximal promoter sequ... | 447 | 12 | 1.98 | 6.20E-07 |
|  |  | 3 | GO:0044212 | transcription regulatory region DNA bind... | 943 | 17 | 4.17 | 6.60E-07 |
|  |  | 4 | GO:0001067 | regulatory region nucleic acid binding | 944 | 17 | 4.18 | 6.70E-07 |
|  |  | 5 | GO:0000987 | proximal promoter sequence-specific DNA ... | 463 | 12 | 2.05 | 8.90E-07 |
|  |  | 6 | GO:0000976 | transcription regulatory region sequence... | 783 | 15 | 3.46 | 1.60E-06 |
|  |  | 7 | GO:0000977 | RNA polymerase II regulatory region sequ... | 697 | 14 | 3.08 | 2.10E-06 |
|  |  | 8 | GO:0001012 | RNA polymerase II regulatory region DNA ... | 700 | 14 | 3.1 | 2.20E-06 |
|  |  | 9 | GO:0003690 | double-stranded DNA binding | 920 | 16 | 4.07 | 2.40E-06 |
|  |  | 10 | GO:0000981 | RNA polymerase II transcription factor a... | 1163 | 18 | 5.14 | 2.70E-06 |
|  |  | 11 | GO:1990837 | sequence-specific double-stranded DNA bi... | 821 | 15 | 3.63 | 2.80E-06 |
|  |  | 12 | GO:0043565 | sequence-specific DNA binding | 1195 | 18 | 5.29 | 3.90E-06 |
|  |  | 13 | GO:0001077 | transcriptional activator activity, RNA ... | 283 | 9 | 1.25 | 4.50E-06 |
|  |  | 14 | GO:0008134 | transcription factor binding | 661 | 13 | 2.92 | 6.40E-06 |
|  |  | 15 | GO:0001228 | transcriptional activator activity, RNA ... | 440 | 10 | 1.95 | 2.40E-05 |
|  |  | 16 | GO:0005515 | protein binding | 13002 | 74 | 57.51 | 4.40E-05 |
|  |  | 17 | GO:0003677 | DNA binding | 2693 | 26 | 11.91 | 6.60E-05 |
|  |  | 18 | GO:0003700 | DNA binding transcription factor activit... | 1646 | 18 | 7.28 | 0.00026 |
|  |  | 19 | GO:0001078 | transcriptional repressor activity, RNA ... | 145 | 5 | 0.64 | 0.00046 |
|  |  | 20 | GO:0140110 | transcription regulator activity | 2041 | 20 | 9.03 | 0.00048 |
|  |  | 21 | GO:0001227 | transcriptional repressor activity, RNA ... | 249 | 6 | 1.1 | 0.00083 |
|  |  | 22 | GO:0042910 | xenobiotic transmembrane transporter act... | 10 | 2 | 0.04 | 0.00085 |
|  |  | 23 | GO:0042975 | peroxisome proliferator activated recept... | 13 | 2 | 0.06 | 0.00146 |
|  |  | 24 | GO:0001222 | transcription corepressor binding | 14 | 2 | 0.06 | 0.0017 |
|  |  | 25 | GO:0005523 | tropomyosin binding | 14 | 2 | 0.06 | 0.0017 |
|  |  | 26 | GO:0004855 | xanthine oxidase activity | 1 | 1 | 0 | 0.00442 |
|  |  | 27 | GO:0005368 | taurine transmembrane transporter activi... | 1 | 1 | 0 | 0.00442 |
|  |  | 28 | GO:0005369 | taurine:sodium symporter activity | 1 | 1 | 0 | 0.00442 |
|  |  | 29 | GO:0016727 | oxidoreductase activity, acting on CH or... | 1 | 1 | 0 | 0.00442 |
|  |  | 30 | GO:0017024 | myosin I binding | 1 | 1 | 0 | 0.00442 |
|  |  | 31 | GO:0031700 | adrenomedullin receptor binding | 1 | 1 | 0 | 0.00442 |
|  |  | 32 | GO:0033142 | progesterone receptor binding | 1 | 1 | 0 | 0.00442 |
|  |  | 33 | GO:0035538 | carbohydrate response element binding | 1 | 1 | 0 | 0.00442 |
|  |  | 34 | GO:0071820 | N-box binding | 1 | 1 | 0 | 0.00442 |
|  |  | 35 | GO:1901363 | heterocyclic compound binding | 6641 | 41 | 29.37 | 0.00644 |
|  |  | 36 | GO:0071889 | 14-3-3 protein binding | 28 | 2 | 0.12 | 0.00678 |
|  |  | 37 | GO:0043014 | alpha-tubulin binding | 29 | 2 | 0.13 | 0.00727 |
|  |  | 38 | GO:0001046 | core promoter sequence-specific DNA bind... | 90 | 3 | 0.4 | 0.00744 |
|  |  | 39 | GO:0046983 | protein dimerization activity | 1386 | 13 | 6.13 | 0.00769 |
|  |  | 40 | GO:0097159 | organic cyclic compound binding | 6732 | 41 | 29.78 | 0.00836 |
|  |  | 41 | GO:0004356 | glutamate-ammonia ligase activity | 2 | 1 | 0.01 | 0.00883 |
|  |  | 42 | GO:0004854 | xanthine dehydrogenase activity | 2 | 1 | 0.01 | 0.00883 |
|  |  | 43 | GO:0016211 | ammonia ligase activity | 2 | 1 | 0.01 | 0.00883 |
|  |  | 44 | GO:0016726 | oxidoreductase activity, acting on CH or... | 2 | 1 | 0.01 | 0.00883 |
|  |  | 45 | GO:0070888 | E-box binding | 37 | 2 | 0.16 | 0.01165 |
|  |  | 46 | GO:0003676 | nucleic acid binding | 4589 | 30 | 20.3 | 0.01183 |
|  |  | 47 | GO:0051087 | chaperone binding | 107 | 3 | 0.47 | 0.01192 |
|  |  | 48 | GO:0004351 | glutamate decarboxylase activity | 3 | 1 | 0.01 | 0.01321 |
|  |  | 49 | GO:0016262 | protein N-acetylglucosaminyltransferase ... | 3 | 1 | 0.01 | 0.01321 |
|  |  | 50 | GO:0016880 | acid-ammonia (or amide) ligase activity | 3 | 1 | 0.01 | 0.01321 |
| Yellow | BP | 1 | GO:0015701 | bicarbonate transport | 44 | 4 | 0.07 | 7.60E-07 |
|  |  | 2 | GO:0015671 | oxygen transport | 15 | 3 | 0.02 | 1.80E-06 |
|  |  | 3 | GO:0015669 | gas transport | 19 | 3 | 0.03 | 3.70E-06 |
|  |  | 4 | GO:0042744 | hydrogen peroxide catabolic process | 24 | 3 | 0.04 | 7.80E-06 |
|  |  | 5 | GO:0042743 | hydrogen peroxide metabolic process | 47 | 3 | 0.08 | 6.10E-05 |
|  |  | 6 | GO:0017001 | antibiotic catabolic process | 50 | 3 | 0.08 | 7.30E-05 |
|  |  | 7 | GO:0051187 | cofactor catabolic process | 59 | 3 | 0.1 | 0.00012 |
|  |  | 8 | GO:0048731 | system development | 5191 | 18 | 8.47 | 0.00034 |
|  |  | 9 | GO:0048856 | anatomical structure development | 6364 | 20 | 10.38 | 0.00045 |
|  |  | 10 | GO:0035239 | tube morphogenesis | 947 | 7 | 1.54 | 0.00069 |
|  |  | 11 | GO:0030282 | bone mineralization | 109 | 3 | 0.18 | 0.00074 |
|  |  | 12 | GO:0098869 | cellular oxidant detoxification | 118 | 3 | 0.19 | 0.00093 |
|  |  | 13 | GO:1990748 | cellular detoxification | 122 | 3 | 0.2 | 0.00102 |
|  |  | 14 | GO:0072593 | reactive oxygen species metabolic proces... | 284 | 4 | 0.46 | 0.00112 |
|  |  | 15 | GO:0032502 | developmental process | 6840 | 20 | 11.15 | 0.00129 |
|  |  | 16 | GO:0048513 | animal organ development | 3789 | 14 | 6.18 | 0.00136 |
|  |  | 17 | GO:0051291 | protein heterooligomerization | 135 | 3 | 0.22 | 0.00136 |
|  |  | 18 | GO:0098754 | detoxification | 139 | 3 | 0.23 | 0.00148 |
|  |  | 19 | GO:0002576 | platelet degranulation | 140 | 3 | 0.23 | 0.00152 |
|  |  | 20 | GO:0042542 | response to hydrogen peroxide | 141 | 3 | 0.23 | 0.00155 |
|  |  | 21 | GO:0007275 | multicellular organism development | 5844 | 18 | 9.53 | 0.0016 |
|  |  | 22 | GO:0016999 | antibiotic metabolic process | 143 | 3 | 0.23 | 0.00161 |
|  |  | 23 | GO:0042737 | drug catabolic process | 143 | 3 | 0.23 | 0.00161 |
|  |  | 24 | GO:0015862 | uridine transport | 1 | 1 | 0 | 0.00163 |
|  |  | 25 | GO:0018166 | C-terminal protein-tyrosinylation | 1 | 1 | 0 | 0.00163 |
|  |  | 26 | GO:0018277 | protein deamination | 1 | 1 | 0 | 0.00163 |
|  |  | 27 | GO:0018322 | protein tyrosinylation | 1 | 1 | 0 | 0.00163 |
|  |  | 28 | GO:1900134 | negative regulation of renin secretion i... | 1 | 1 | 0 | 0.00163 |
|  |  | 29 | GO:1904073 | regulation of trophectodermal cell proli... | 1 | 1 | 0 | 0.00163 |
|  |  | 30 | GO:1904075 | positive regulation of trophectodermal c... | 1 | 1 | 0 | 0.00163 |
|  |  | 31 | GO:0031214 | biomineral tissue development | 145 | 3 | 0.24 | 0.00168 |
|  |  | 32 | GO:0051281 | positive regulation of release of seques... | 38 | 2 | 0.06 | 0.00175 |
|  |  | 33 | GO:0048468 | cell development | 2201 | 10 | 3.59 | 0.00194 |
|  |  | 34 | GO:0048009 | insulin-like growth factor receptor sign... | 41 | 2 | 0.07 | 0.00203 |
|  |  | 35 | GO:0034762 | regulation of transmembrane transport | 564 | 5 | 0.92 | 0.00204 |
|  |  | 36 | GO:0035295 | tube development | 1141 | 7 | 1.86 | 0.00206 |
|  |  | 37 | GO:0072359 | circulatory system development | 1155 | 7 | 1.88 | 0.00221 |
|  |  | 38 | GO:0060562 | epithelial tube morphogenesis | 363 | 4 | 0.59 | 0.00275 |
|  |  | 39 | GO:0050770 | regulation of axonogenesis | 176 | 3 | 0.29 | 0.00291 |
|  |  | 40 | GO:0046579 | positive regulation of Ras protein signa... | 51 | 2 | 0.08 | 0.00313 |
|  |  | 41 | GO:0009994 | oocyte differentiation | 52 | 2 | 0.08 | 0.00325 |
|  |  | 42 | GO:0010524 | positive regulation of calcium ion trans... | 52 | 2 | 0.08 | 0.00325 |
|  |  | 43 | GO:0035904 | aorta development | 52 | 2 | 0.08 | 0.00325 |
|  |  | 44 | GO:0001834 | trophectodermal cell proliferation | 2 | 1 | 0 | 0.00326 |
|  |  | 45 | GO:0007529 | establishment of synaptic specificity at... | 2 | 1 | 0 | 0.00326 |
|  |  | 46 | GO:0015864 | pyrimidine nucleoside transport | 2 | 1 | 0 | 0.00326 |
|  |  | 47 | GO:0030334 | regulation of cell migration | 917 | 6 | 1.5 | 0.00327 |
|  |  | 48 | GO:0120035 | regulation of plasma membrane bounded ce... | 631 | 5 | 1.03 | 0.00332 |
|  |  | 49 | GO:0046626 | regulation of insulin receptor signaling... | 53 | 2 | 0.09 | 0.00337 |
|  |  | 50 | GO:0031344 | regulation of cell projection organizati... | 641 | 5 | 1.05 | 0.00355 |
|  | CC | 1 | GO:0031838 | haptoglobin-hemoglobin complex | 4 | 3 | 0.01 | 1.80E-08 |
|  |  | 2 | GO:0005833 | hemoglobin complex | 12 | 3 | 0.02 | 9.60E-07 |
|  |  | 3 | GO:0071682 | endocytic vesicle lumen | 20 | 3 | 0.03 | 4.90E-06 |
|  |  | 4 | GO:0005576 | extracellular region | 5443 | 21 | 9.18 | 2.60E-05 |
|  |  | 5 | GO:0060205 | cytoplasmic vesicle lumen | 392 | 6 | 0.66 | 4.50E-05 |
|  |  | 6 | GO:0031983 | vesicle lumen | 393 | 6 | 0.66 | 4.50E-05 |
|  |  | 7 | GO:0072562 | blood microparticle | 152 | 4 | 0.26 | 0.00012 |
|  |  | 8 | GO:0031089 | platelet dense granule lumen | 14 | 2 | 0.02 | 0.00025 |
|  |  | 9 | GO:0042827 | platelet dense granule | 21 | 2 | 0.04 | 0.00057 |
|  |  | 10 | GO:0005887 | integral component of plasma membrane | 2106 | 10 | 3.55 | 0.00192 |
|  |  | 11 | GO:0045121 | membrane raft | 336 | 4 | 0.57 | 0.00238 |
|  |  | 12 | GO:0098857 | membrane microdomain | 337 | 4 | 0.57 | 0.0024 |
|  |  | 13 | GO:0031226 | intrinsic component of plasma membrane | 2174 | 10 | 3.67 | 0.00244 |
|  |  | 14 | GO:0098589 | membrane region | 348 | 4 | 0.59 | 0.0027 |
|  |  | 15 | GO:0005615 | extracellular space | 4339 | 15 | 7.32 | 0.00275 |
|  |  | 16 | GO:0044421 | extracellular region part | 4578 | 15 | 7.72 | 0.00473 |
|  |  | 17 | GO:0022627 | cytosolic small ribosomal subunit | 63 | 2 | 0.11 | 0.00506 |
|  |  | 18 | GO:0035867 | alphav-beta3 integrin-IGF-1-IGF1R comple... | 4 | 1 | 0.01 | 0.00673 |
|  |  | 19 | GO:0042567 | insulin-like growth factor ternary compl... | 4 | 1 | 0.01 | 0.00673 |
|  |  | 20 | GO:0016942 | insulin-like growth factor binding prote... | 5 | 1 | 0.01 | 0.0084 |
|  |  | 21 | GO:0036454 | growth factor complex | 5 | 1 | 0.01 | 0.0084 |
|  |  | 22 | GO:0070062 | extracellular exosome | 3105 | 11 | 5.24 | 0.01069 |
|  |  | 23 | GO:0031012 | extracellular matrix | 519 | 4 | 0.88 | 0.01095 |
|  |  | 24 | GO:1903561 | extracellular vesicle | 3127 | 11 | 5.27 | 0.01126 |
|  |  | 25 | GO:0043230 | extracellular organelle | 3129 | 11 | 5.28 | 0.01131 |
|  |  | 26 | GO:0015935 | small ribosomal subunit | 96 | 2 | 0.16 | 0.0114 |
|  |  | 27 | GO:0001652 | granular component | 7 | 1 | 0.01 | 0.01174 |
|  |  | 28 | GO:0044445 | cytosolic part | 287 | 3 | 0.48 | 0.01235 |
|  |  | 29 | GO:0098590 | plasma membrane region | 1170 | 6 | 1.97 | 0.0126 |
|  |  | 30 | GO:0036056 | filtration diaphragm | 8 | 1 | 0.01 | 0.01341 |
|  |  | 31 | GO:0036057 | slit diaphragm | 8 | 1 | 0.01 | 0.01341 |
|  |  | 32 | GO:0002116 | semaphorin receptor complex | 11 | 1 | 0.02 | 0.0184 |
|  |  | 33 | GO:0031094 | platelet dense tubular network | 11 | 1 | 0.02 | 0.0184 |
|  |  | 34 | GO:0016324 | apical plasma membrane | 335 | 3 | 0.56 | 0.01862 |
|  |  | 35 | GO:0030141 | secretory granule | 959 | 5 | 1.62 | 0.02139 |
|  |  | 36 | GO:0030018 | Z disc | 134 | 2 | 0.23 | 0.02142 |
|  |  | 37 | GO:0043020 | NADPH oxidase complex | 13 | 1 | 0.02 | 0.02171 |
|  |  | 38 | GO:0044459 | plasma membrane part | 3442 | 11 | 5.8 | 0.02228 |
|  |  | 39 | GO:0022626 | cytosolic ribosome | 138 | 2 | 0.23 | 0.02262 |
|  |  | 40 | GO:0031253 | cell projection membrane | 365 | 3 | 0.62 | 0.02329 |
|  |  | 41 | GO:0031982 | vesicle | 4920 | 14 | 8.3 | 0.02347 |
|  |  | 42 | GO:0034774 | secretory granule lumen | 374 | 3 | 0.63 | 0.02481 |
|  |  | 43 | GO:0031674 | I band | 147 | 2 | 0.25 | 0.02544 |
|  |  | 44 | GO:0031410 | cytoplasmic vesicle | 2636 | 9 | 4.44 | 0.02743 |
|  |  | 45 | GO:0097708 | intracellular vesicle | 2639 | 9 | 4.45 | 0.02761 |
|  |  | 46 | GO:0031362 | anchored component of external side of p... | 18 | 1 | 0.03 | 0.02993 |
|  |  | 47 | GO:0045177 | apical part of cell | 404 | 3 | 0.68 | 0.03024 |
|  |  | 48 | GO:0043034 | costamere | 19 | 1 | 0.03 | 0.03157 |
|  |  | 49 | GO:0031252 | cell leading edge | 411 | 3 | 0.69 | 0.03159 |
|  |  | 50 | GO:0030139 | endocytic vesicle | 414 | 3 | 0.7 | 0.03218 |
|  | MF | 1 | GO:0031720 | haptoglobin binding | 3 | 3 | 0.01 | 4.90E-09 |
|  |  | 2 | GO:0005344 | oxygen carrier activity | 14 | 3 | 0.02 | 1.80E-06 |
|  |  | 3 | GO:0140104 | molecular carrier activity | 43 | 3 | 0.08 | 5.70E-05 |
|  |  | 4 | GO:0004601 | peroxidase activity | 49 | 3 | 0.09 | 8.50E-05 |
|  |  | 5 | GO:0019825 | oxygen binding | 51 | 3 | 0.09 | 9.60E-05 |
|  |  | 6 | GO:0016684 | oxidoreductase activity, acting on perox... | 54 | 3 | 0.09 | 0.00011 |
|  |  | 7 | GO:0030506 | ankyrin binding | 20 | 2 | 0.03 | 0.00055 |
|  |  | 8 | GO:0016209 | antioxidant activity | 99 | 3 | 0.17 | 0.00068 |
|  |  | 9 | GO:0005158 | insulin receptor binding | 25 | 2 | 0.04 | 0.00087 |
|  |  | 10 | GO:0004835 | tubulin-tyrosine ligase activity | 1 | 1 | 0 | 0.00175 |
|  |  | 11 | GO:0004900 | erythropoietin receptor activity | 1 | 1 | 0 | 0.00175 |
|  |  | 12 | GO:0036143 | kringle domain binding | 1 | 1 | 0 | 0.00175 |
|  |  | 13 | GO:0020037 | heme binding | 143 | 3 | 0.25 | 0.00197 |
|  |  | 14 | GO:0046906 | tetrapyrrole binding | 153 | 3 | 0.27 | 0.00239 |
|  |  | 15 | GO:0016491 | oxidoreductase activity | 810 | 6 | 1.42 | 0.00254 |
|  |  | 16 | GO:0005506 | iron ion binding | 173 | 3 | 0.3 | 0.00338 |
|  |  | 17 | GO:0008073 | ornithine decarboxylase inhibitor activi... | 3 | 1 | 0.01 | 0.00524 |
|  |  | 18 | GO:0004605 | phosphatidate cytidylyltransferase activ... | 4 | 1 | 0.01 | 0.00698 |
|  |  | 19 | GO:0044323 | retinoic acid-responsive element binding | 4 | 1 | 0.01 | 0.00698 |
|  |  | 20 | GO:0005134 | interleukin-2 receptor binding | 5 | 1 | 0.01 | 0.00871 |
|  |  | 21 | GO:0015057 | thrombin-activated receptor activity | 5 | 1 | 0.01 | 0.00871 |
|  |  | 22 | GO:0030492 | hemoglobin binding | 5 | 1 | 0.01 | 0.00871 |
|  |  | 23 | GO:0004720 | protein-lysine 6-oxidase activity | 6 | 1 | 0.01 | 0.01045 |
|  |  | 24 | GO:0042979 | ornithine decarboxylase regulator activi... | 6 | 1 | 0.01 | 0.01045 |
|  |  | 25 | GO:0004896 | cytokine receptor activity | 94 | 2 | 0.16 | 0.01173 |
|  |  | 26 | GO:0005337 | nucleoside transmembrane transporter act... | 7 | 1 | 0.01 | 0.01218 |
|  |  | 27 | GO:0034235 | GPI anchor binding | 7 | 1 | 0.01 | 0.01218 |
|  |  | 28 | GO:0008510 | sodium:bicarbonate symporter activity | 9 | 1 | 0.02 | 0.01563 |
|  |  | 29 | GO:0031681 | G-protein beta-subunit binding | 9 | 1 | 0.02 | 0.01563 |
|  |  | 30 | GO:0070567 | cytidylyltransferase activity | 10 | 1 | 0.02 | 0.01735 |
|  |  | 31 | GO:0016175 | superoxide-generating NADPH oxidase acti... | 11 | 1 | 0.02 | 0.01907 |
|  |  | 32 | GO:0016176 | superoxide-generating NADPH oxidase acti... | 11 | 1 | 0.02 | 0.01907 |
|  |  | 33 | GO:0017154 | semaphorin receptor activity | 11 | 1 | 0.02 | 0.01907 |
|  |  | 34 | GO:0043495 | protein membrane anchor | 12 | 1 | 0.02 | 0.02079 |
|  |  | 35 | GO:0005178 | integrin binding | 129 | 2 | 0.23 | 0.02133 |
|  |  | 36 | GO:0050664 | oxidoreductase activity, acting on NAD(P... | 16 | 1 | 0.03 | 0.02763 |
|  |  | 37 | GO:0016500 | protein-hormone receptor activity | 17 | 1 | 0.03 | 0.02933 |
|  |  | 38 | GO:0016641 | oxidoreductase activity, acting on the C... | 17 | 1 | 0.03 | 0.02933 |
|  |  | 39 | GO:0030546 | receptor activator activity | 17 | 1 | 0.03 | 0.02933 |
|  |  | 40 | GO:0004745 | retinol dehydrogenase activity | 18 | 1 | 0.03 | 0.03103 |
|  |  | 41 | GO:0005159 | insulin-like growth factor receptor bind... | 18 | 1 | 0.03 | 0.03103 |
|  |  | 42 | GO:0051861 | glycolipid binding | 19 | 1 | 0.03 | 0.03272 |
|  |  | 43 | GO:0052650 | NADP-retinol dehydrogenase activity | 19 | 1 | 0.03 | 0.03272 |
|  |  | 44 | GO:0005102 | signaling receptor binding | 1810 | 7 | 3.17 | 0.03442 |
|  |  | 45 | GO:0001965 | G-protein alpha-subunit binding | 21 | 1 | 0.04 | 0.03611 |
|  |  | 46 | GO:0016881 | acid-amino acid ligase activity | 21 | 1 | 0.04 | 0.03611 |
|  |  | 47 | GO:0015106 | bicarbonate transmembrane transporter ac... | 23 | 1 | 0.04 | 0.03948 |
|  |  | 48 | GO:0016638 | oxidoreductase activity, acting on the C... | 23 | 1 | 0.04 | 0.03948 |
|  |  | 49 | GO:0043325 | phosphatidylinositol-3,4-bisphosphate bi... | 26 | 1 | 0.05 | 0.04451 |
|  |  | 50 | GO:0043236 | laminin binding | 29 | 1 | 0.05 | 0.04952 |
