# Supplementary material for: Transcriptional profiling to identify the key genes and pathways of pterygium
Source: PeerJ. 2020 May 4;8:e9056. doi: 10.7717/peerj.9056 (PMC7204871; doi:10.7717/peerj.9056)
Supplement: Table S4 [file peerj-08-9056-s004.docx]

**Supplementary table 4 Genes significantly perturbed in each single subject with PEEP method**

| **Case number** | **Gene ID** |
| --- | --- |
| X1 | 23129 952 6521 9703 8522 7005 30845 3479 23187 3631 10863 4052 2006 27122 5318 9697 259266 51208 26153 221395 489 54431 5788 2950 23259 23645 2036 393 30835 3655 151887 7051 51816 54873 6523 5641 23613 8760 9388 25878 688 23406 80381 29997 57664 10430 857 2887 6424 9827 8395 573 6387 81855 1277 23220 6484 7450 9761 4256 79794 2026 2030 3910 10279 6678 1809 604 150465 2976 55704 2335 6382 5334 2034 2152 3664 23120 5918 894 64207 4053 57718 157506 10237 5737 10268 8434 57670 80114 6590 56937 7499 1948 5787 146330 83460 5420 2318 57045 4016 6665 65095 9459 7140 6533 84951 79883 3021 10631 4629 694 8553 377 187 9053 2752 10154 6326 1515 22801 4091 10579 8549 51228 80816 2627 79755 2886 79180 3339 81607 83872 1893 287 10788 84466 340351 357 79742 114569 84985 85449 83706 84159 11098 285313 283349 7070 58494 7071 5799 57188 7102 114907 2114 116987 7498 10163 212 57519 1636 10417 51673 808 3275 51149 255877 9249 22854 92291 2487 7123 51705 1278 1536 116159 8642 58475 10982 2021 7846 29946 64359 11259 1281 25849 4330 1942 5724 51621 3866 11076 3860 131578 9451 10231 53342 54961 26579 57493 947 53833 8322 1466 7025 2 379 84747 55057 9240 2907 1960 91663 4947 8347 2149 633 11248 1436 144608 10082 8338 6586 8349 4588 3850 11118 2057 79875 7373 10123 643853 3040 54510 284340 2526 64499 6840 1803 1438 27347 22899 7052 340527 6920 3039 81606 54492 100505664 79940 387882 3043 10628 9294 100462981 11165 8362 8343 |
| X2 | 2006 51208 26153 51816 9388 1628 1277 10279 604 150465 2976 57718 60370 8434 7499 9572 83460 7538 57045 65095 79883 10631 694 22801 8549 340351 85449 5799 7102 212 51673 9249 22854 2487 1999 58475 10982 29946 1942 131578 26579 8322 379 9240 1960 91663 10082 8338 4588 11118 3040 27347 3039 1052 79940 3043 |
| X4 | 952 9703 8522 30845 23187 2006 54431 3655 151887 51816 5641 8760 25878 688 29108 80381 29997 1628 857 2887 6424 573 23220 7450 9761 79794 10279 1809 55704 2335 6382 894 64207 4053 57718 10237 10268 4246 83460 5420 57045 4016 6665 79883 9053 8549 51228 79180 3339 83872 1893 84466 79742 11098 285313 58494 7102 116987 10163 212 51673 808 3933 51705 2021 29946 3008 25849 5724 51621 11076 9451 10231 54961 57493 84747 1960 91663 1436 144608 6586 4588 10123 54510 6920 54492 387882 9294 102724428 8343 |
| X5 | 5318 23645 4616 688 23581 29997 10430 573 81855 604 3280 2152 3664 5918 60370 1843 157506 57670 6590 56937 1948 7538 3021 694 2752 10579 79755 150094 5799 1636 255877 81788 22829 116159 58475 64359 3008 4330 51621 3726 7025 9240 2907 247 10082 8338 79875 3006 10123 643338 2526 1438 57126 81606 1052 100505664 10628 102724428 |
| X6 | 23129 9703 7005 23187 10863 4052 9697 26153 5788 23259 393 30835 7051 9388 80381 10430 9827 1277 2030 3910 6678 1809 150465 2034 23120 894 10237 5787 4016 187 1893 287 340351 357 83706 84159 7070 58494 7071 2114 57519 92291 1278 1536 8642 2021 1281 53342 57493 1466 7025 247 2149 633 642273 4588 64499 6840 22899 7052 340527 387882 654790 |
| X7 | 23613 1628 57664 2976 9572 57045 65095 9053 25833 10579 2886 3339 51149 22854 1999 54961 27347 8362 |
| X8 | 259266 221395 54873 23406 6424 8395 4256 8434 80114 5420 9459 22801 80816 2627 10788 85449 283349 7123 10982 131578 947 8322 11248 2057 7373 3040 54510 3039 79940 3043 |
| X9 | 489 10631 10788 2487 53342 |
| X10 | 6521 9703 51085 3479 3631 4052 27122 5318 9697 259266 51208 26153 221395 489 54431 23259 393 3655 6523 9388 23406 1628 10430 857 2887 6424 9827 8395 6387 81855 23220 7450 9761 4256 79794 2026 2030 10279 604 2335 26270 894 57718 10237 5737 57670 56937 7499 1948 9572 5787 83460 7538 5420 2318 57045 65095 79883 187 6326 4091 8549 2627 79180 83872 1893 10788 84466 340351 79742 114569 283349 5799 57188 116987 212 1636 808 3275 255877 22854 116159 58475 2021 64359 5724 131578 10231 53342 26579 379 84747 9240 1960 91663 144608 10082 4588 2057 7373 643853 3040 2526 1803 1438 3039 54492 387882 3043 100462981 8362 |
| X13 | 23129 6521 4052 2006 27122 5318 9697 51208 26153 221395 5788 23259 151887 51816 54873 6523 5641 23406 857 6424 6387 1277 7450 4256 2026 10279 6678 3280 2335 5334 26270 894 4053 10237 5737 10268 8434 57670 80114 56937 7499 9572 5787 83460 7538 2318 57045 9459 79883 10631 187 10154 1515 22801 4091 8549 2627 83872 1893 287 10788 84466 79742 114569 85449 83706 11098 283349 7070 58494 57188 7102 116987 212 10417 51673 808 255877 22854 1999 7123 51705 1536 8642 58475 7846 11259 1281 25849 4330 1942 5724 51621 131578 10231 53342 947 8322 7025 2 379 84747 9240 91663 2149 633 11248 1436 144608 10082 8338 6586 3850 11118 2057 79875 7373 3040 54510 64499 1803 1438 22899 3039 81606 54492 1052 79940 387882 3043 9294 100462981 11165 8362 |
| X14 | 6521 3479 51208 26153 489 2950 30835 9388 29997 2887 6424 8395 1277 604 3280 2976 2335 26270 3664 64207 57718 5737 9572 7538 2318 4016 65095 7140 79883 694 8553 9053 1515 25833 10579 8549 2886 357 85449 7070 5799 212 51149 22854 1999 7123 1536 58475 29946 64359 1942 131578 9451 53342 54961 26579 7025 379 9240 1960 91663 8347 2149 11248 10082 8338 4588 3850 2057 643853 3040 64499 1438 27347 3039 1052 3043 8362 8343 |
| X15 | 23129 952 6521 3479 3631 4052 2006 9697 259266 51208 26153 489 5788 393 23613 80381 10430 2887 6424 9827 6387 7450 2026 2976 5334 4053 57718 157506 5737 10268 57670 56937 1948 9572 5787 83460 5420 2318 4016 9459 79883 187 10154 25833 22801 4091 79180 3339 1893 84466 340351 79742 85449 83706 58494 57188 116987 212 10417 51673 808 2487 7123 116159 8642 58475 2021 29946 4330 1942 5724 51621 11076 131578 10231 8322 7025 9240 91663 8347 2149 633 11248 6586 4588 3850 2057 643853 3040 2526 64499 1803 1438 27347 22899 3039 54492 79940 3043 9294 100462981 8362 |
| X16 | 10863 259266 221395 7499 187 7455 7498 9249 1056 3860 26579 55057 54101 283131 |
| X17 | 2950 5334 57045 694 114569 5799 22854 58475 1942 379 8347 8338 8349 11118 6920 11165 8343 |
| X18 | 4057 3631 4616 8760 2026 3007 4246 4629 1515 3933 92291 |
| X19 | 51085 27122 10397 6513 9572 146330 79883 114569 133 114907 379 374907 3850 8362 |
| X20 | 23129 952 6521 9703 8522 7005 3479 23187 3631 4052 2006 27122 5318 9697 259266 51208 26153 221395 489 54431 5788 23259 393 30835 3655 151887 51816 54873 6523 5641 8760 25878 23406 29108 80381 57664 10430 857 2887 6424 9827 8395 6387 81855 1277 23220 7450 9761 4256 79794 2026 2030 3910 10279 6678 1809 150465 2976 55704 2335 5334 3664 23120 894 64207 4053 157506 10237 5737 10268 8434 57670 80114 56937 7499 1948 5787 83460 5420 2318 57045 4016 65095 9459 6533 79883 10631 694 377 187 10154 6326 1515 22801 4091 8549 51228 2627 79180 3339 81607 83872 1893 287 10788 84466 340351 79742 114569 85449 83706 11098 285313 283349 7070 58494 57188 7102 114907 116987 10163 212 57519 1636 10417 51673 808 3275 51149 255877 9249 2487 7123 51705 1278 1536 116159 8642 58475 10982 2021 7846 29946 64359 11259 1281 25849 4330 1942 5724 51621 11076 131578 10231 53342 57493 947 8322 7025 2 84747 9240 5187 91663 4947 2149 633 11248 1436 144608 10082 6586 4588 11118 2057 79875 7373 643853 3040 54510 2526 64499 6840 1438 22899 340527 3039 81606 54492 79940 387882 3043 9294 100462981 11165 8362 |
| X21 | 23129 952 6521 9703 8522 3479 23187 3631 4052 2006 27122 5318 9697 259266 51208 26153 221395 489 54431 5788 23259 393 30835 3655 151887 51816 54873 6523 5641 8760 9388 25878 23406 80381 57664 10430 857 2887 6424 9827 8395 6387 81855 1277 23220 7450 9761 4256 79794 2026 2030 3910 10279 6678 1809 150465 2976 55704 2335 5334 26270 894 4053 157506 10237 5737 10268 8434 57670 80114 56937 7499 1948 9572 5787 83460 5420 2318 57045 4016 9459 79883 10631 694 377 187 10154 6326 1515 22801 4091 8549 51228 2627 79180 3339 83872 1893 287 10788 84466 340351 79742 114569 85449 83706 11098 285313 283349 7070 58494 57188 7102 116987 10163 212 57519 1636 10417 51673 808 3275 255877 2487 7123 51705 1278 1536 116159 8642 58475 10982 2021 7846 11259 1281 25849 4330 5724 51621 11076 131578 9451 10231 53342 26579 57493 947 8322 7025 2 84747 9240 1960 91663 4947 2149 633 11248 1436 144608 10082 6586 4588 11118 2057 79875 7373 643853 3040 54510 2526 64499 1803 1438 22899 340527 3039 81606 54492 79940 387882 3043 9294 100462981 11165 8362 |
